# Automatic segmentation of skeletal muscles from MR images using modified U-Net and a novel data augmentation approach

**DOI:** 10.3389/fbioe.2024.1355735

**Published:** 2024-02-22

**Authors:** Zhicheng Lin, William H. Henson, Lisa Dowling, Jennifer Walsh, Enrico Dall’Ara, Lingzhong Guo

**Affiliations:** ^1^ Department of Automatic Control and Systems Engineering, University of Sheffield, Sheffield, United Kingdom; ^2^ Department of Mechanical Engineering, University of Sheffield, Sheffield, United Kingdom; ^3^ Faculty of Health, University of Sheffield, Sheffield, United Kingdom; ^4^ Department of Oncology and Metabolism, University of Sheffield, Sheffield, United Kingdom; ^5^ Insigneo, University of Sheffield, Sheffield, United Kingdom

**Keywords:** muscle, neural network, deep learning, automatic segmentation, data augmentation

## Abstract

Rapid and accurate muscle segmentation is essential for the diagnosis and monitoring of many musculoskeletal diseases. As gold standard, manual annotation suffers from intensive labor and high inter-operator reproducibility errors. In this study, deep learning (DL) based automatic muscle segmentation from MR scans is investigated for post-menopausal women, who normally experience a decline in muscle volume. The performance of four Deep Learning (DL) models was evaluated: U-Net and UNet++ and two modified U-Net networks, which combined feature fusion and attention mechanisms (Feature-Fusion-UNet, FFU, and Attention-Feature-Fusion-UNet, AFFU). The models were tested for automatic segmentation of 16-lower limb muscles from MRI scans of two cohorts of post-menopausal women (11 subjects in PMW-1, 8 subjects in PMW-2; from two different studies so considered independent datasets) and 10 obese post-menopausal women (PMW-OB). Furthermore, a novel data augmentation approach is proposed to enlarge the training dataset. The results were assessed and compared by using the Dice similarity coefficient (DSC), relative volume error (RVE), and Hausdorff distance (HD). The best performance among all four DL models was achieved by AFFU (PMW-1: DSC 0.828 ± 0.079, 1-RVE 0.859 ± 0.122, HD 29.9 mm ± 26.5 mm; PMW-2: DSC 0.833 ± 0.065, 1-RVE 0.873 ± 0.105, HD 25.9 mm ± 27.9 mm; PMW-OB: DSC 0.862 ± 0.048, 1-RVE 0.919 ± 0.076, HD 34.8 mm ± 46.8 mm). Furthermore, the augmentation of data significantly improved the DSC scores of U-Net and AFFU for all 16 tested muscles (between 0.23% and 2.17% (DSC), 1.6%–1.93% (1-RVE), and 9.6%–19.8% (HD) improvement). These findings highlight the feasibility of utilizing DL models for automatic segmentation of muscles in post-menopausal women and indicate that the proposed augmentation method can enhance the performance of models trained on small datasets.

## 1 Introduction

Human muscles, particularly the skeletal muscles of the lower limbs, play an indispensable role in generating strength and facilitating movement in daily life (Pandy and Andriacchi, 2010). Some diseases or chronic conditions, such as age-related loss of muscle mass and strength (e.g., sarcopenia), can result in impaired mobility (Cruz-Jentoft et al., 2010). Stroke patients (Nishioka et al., 2021) often experience sarcopenia due to denervation, local inflammation, and inadequate nutrient intake, leading to loss of skeletal muscle mass. Neurological disorders, such as cerebral palsy (D’Souza et al., 2019), caused by non-progressive brain injury at birth can also result in musculoskeletal deformities in children. The assessment of changes of muscle properties over time can enable a better understanding of the effect of musculoskeletal diseases and provide more effective diagnosis approaches and interventions (Krzysztofik et al., 2019). Nevertheless, there are still challenges in effective assessment of muscle properties from the clinical biomedical images (Computed Tomography, CT, or Magnetic Resonance Imaging, MRI).

CT and MRI have been widely used to measure muscle volume or shape, with MRI being more popular as it does not induce ionizing radiation to the patient (Montefiori et al., 2020; Verdú-Díaz et al., 2020). Muscle segmentation is one of the fundamental techniques used to measure the geometrical properties of skeletal muscles that can be associated with the structural functional properties of muscles (Lareau-Trudel et al., 2015; Montefiori et al., 2020; Zhu et al., 2021). Currently, the common method for measuring relevant muscle characteristics is manual segmentation of relevant areas of interest on MRI slices (Montefiori et al., 2020; Davico et al., 2022). However, manual segmentation takes approximately 10 hours for an experienced operator to process thirty-five lower limb muscles and is associated with low inter-operator reproducibility for some of the muscles, with variation above 10% (Montefiori et al., 2020). Over the past decades, many image segmentation methods have been proposed, which are generally based on digital image processing coupled with optimization algorithms. These traditional segmentation methods (Otsu, 1979; Adams and Bischof, 1994; Helen et al., 2011) normally extract low-level semantics of images, such as color, texture, or shape information, without considering high-level semantic information, and could lead to imprecise labeling results. With the emergence of Deep Learning (DL), Artificial Neural Networks (ANNs), and Convolutional Neural Networks (CNNs) (Simonyan and Zisserman, 2015; Krizhevsky et al., 2017), traditional segmentation methods are gradually being replaced since semantic segmentation algorithms based on DL and CNNs can extract mid-to-high-level semantic information from images, improving the segmentation results (Tao et al., 2018).

In recent years, some DL based muscle segmentation methods have been proposed and demonstrated promising prospects for replacing manual segmentation. Zhu et al. (Zhu et al., 2021) presented a hybrid model based on basic U-Net (Ronneberger, Fischer, and Brox, 2015) that achieved an average DSC of 0.88 in 11 lower limb muscles of 20 healthy children and children with cerebral palsy (Ni et al., 2019). proposed a 3D CNN that showed similar performance to manual segmentation in all lower limb individual muscles of young athletes (*n* = 64), achieving a Dice similarity coefficient (DSC) of approximately 0.9. However, muscle automatic segmentation is currently limited with respect to subject range and there are no results about post-menopausal or obese women in the literature. In particular, compared to children and young men, post-menopausal women experience a decline in muscle content with aging, resulting in less distinct boundaries between individual muscles and an increase in fat content, which poses a greater challenge for automatic individual muscle segmentation. Moreover, the segmentation of skeletal muscles in post-menopausal obese women is potentially more challenging due to the thick layer of fat around the muscles. While automatic image segmentation approaches based on CNN algorithms are usually calibrated and tested on similar cohorts of subjects, in order to improve the applicability of the models it would be beneficial to assess the model’s accuracy for different testing datasets.

Currently, one of the most popular DL networks used for medical image segmentation is U-Net (Zhu et al., 2021) and has been shown to be powerful in medical image segmentation, e.g., cell segmentation (Ronneberger et al., 2015), liver and tumor segmentation (Kushnure and Talbar, 2021). Despite the success of existing U-Net type networks, they suffer from some limitations including the hard coding of the receptive field size, as well as that they do not account for inherent noise in the data. Several improved U-Net networks have been proposed, where UNet++ (Zhou et al., 2018), an improved version of U-Net, employs multiple short connections to enhance the integration of different receptive fields at each layer, compromising increasing computational cost. In (Ibtehaz and Sohel Rahman, 2020), the MultiResUNet was proposed to enhance the semantic integration between the encoder and decoder; however, it only focuses the integration within the same layer. In the expansion path of the U-Net architecture, deep features are gradually combined with shallow features from bottom to top. This process allows the fusion of semantic elements from adjacent layers, but as moving towards the upper layers, there remains a semantic gap between non-adjacent layers, resulting in information loss. To address this and improve the integration of semantic features from different layers, in this study modified U-Net networks have been developed, by adopting feature fusion and attention mechanisms (Oktay et al., 2018; Woo et al., 2018).

One issue usually associated with supervised learning for image segmentation is the limited size of the training image dataset, which can have a significant impact on the performance of automatic segmentation methods (Hesamian et al., 2019). Data augmentation methods including random scaling, cropping, rotation, flipping, and adding noise as well as spatial and grayscale transformations are normally used to overcome the limitation of data size (Krizhevsky et al., 2017; Jackson et al., 2019). Compared with natural object standard image augmentation, it is more challenging for the augmentation of medical images because of the stricter requirements of the medical and/or anatomical significance. There has been little research on MRI image augmentation methods. Thyreau et al., 2018 segmented the hippocampus by modifying the image border’s contrast, while Ronneberger et al., 2015; Dong et al., 2017 used deformable registration to segment brain tumors and cells, respectively. Shin et al., 2018 synthesized abnormal brain tumor MRI images using GAN (Generative Adversarial Networks) networks for data augmentation and optimized model performance. However, these methods have limitations associated with the segmentation of individual muscles. In fact, deformable image registration may excessively deform the muscles’ shape in new subjects, leading to a loss of anatomical consistency (i.e., the spatial location, size, and shape of the muscles) (Henson et al., 2023a).

Therefore, the aim of this study was to develop new DL methods for the automatic segmentation of most individual skeletal muscles in the lower limb from MR imaging data of postmenopausal women and propose a novel data augmentation approach based on SSM (Statistical Shape Model) for increasing the size of the MRI datasets (Hesamian et al., 2019; Henson et al., 2023b).

## 2 Materials and methods

### 2.1 Participants

The data from three different cohorts of post-menopausal women (PMW) were utilized in this study. The first cohort consisted of T1-weighted magnetic resonance images (MR images) of 11 PMW (PMW-1, age 69.0 ± 6.7 years old; weight 66.9 ± 7.7 kg; height 159 ± 3 cm; BMI: 26.5 ± 3.4) with no muscle disease who were recruited by the Metabolic Bone Centre (Sheffield, UK) as part of larger studies (approved by the East of England–Cambridgeshire and Hertfordshire Research Ethics Committee and the Health Research Authority, Reference 16/EE/0049) (Montefiori et al., 2020). Two other cohorts recruited from a previous observational (approved by the Leeds West Research Ethics Committee, Reference 20/YH/0274) study were included in this work: 8 PMW with no muscle diseases (PMW-2, age 65.8 ± 3.9 years old; weight 57.1 ± 5.8 kg; height 161 ± 3 cm; BMI: 22.0 ± 2.1) and 10 obese PMW (PMW-OB, age 64.7 ± 4.5 years old; weight 84.1 ± 15.6 kg; height 159 ± 9 cm; BMI: 33.0 ± 3.2) (Lisa, 2023). Although the two cohorts were imaged in two different studies, the scans were performed in the same hospital by using two different 1.5T MRI scanners, with the same scanning protocol as described below.

All the full lower-limb MRI scans were performed on the 1.5T Siemens Magnetom Avanto or 1.5T Siemens Magnetom Aera (Siemens AG, Erlangen, Germany). Four sequences were taken to capture the hip, thigh, knee, and shank (Henson et al., 2023a). To minimize scanning time without losing detailed joint geometries the joints were scanned with a higher resolution (pixel size of 1.05 mm × 1.05 mm and slice thickness of 3.00 mm) than the central portions of the long bones (pixel size of 1.15 mm × 1.15 mm and slice thickness of 5.00 mm). The sequences were then combined in MATLAB (R2006a) to create a continuous 3D image from the hip to the ankle (Henson et al., 2023b). This was done by standardizing the resolution of each imaging sequence from the different sections through tri-linear interpolation (interp3, MATLAB R2006a) to be 1.00 mm × 1.00 mm × 1.00 mm. In this study, a total of 25 lower limb muscles spanning from the knee to the hip were manually segmented by three operators (PhD students or Postdoctoral researchers who have been trained on at least 15 datasets) for each subject, which is considered the gold standard for muscle segmentation.

The inter- and intra-operator reproducibility of the manual segmentation was found to differ from muscles to muscles (range CoV: 4.2%–22.8%) (Montefiori et al., 2020). and only some of them were reproducible enough to be used for calibrating the DL models. Ultimately, during the model evaluation phase, 16 muscles were selected as the objective muscles for analysis: rectus femoris (RF), vastus intermedius (VI), vastus lateralis (VL), vastus medialis (VM), sartorius (SAT), semimembranosus (SMB), semitendinosus (SMT), gracilis (GRA), biceps femoris caput brevis (BCB), biceps femoris caput longum (BCL), adductor magnus (AM), adductor brevis (AB), adductor longus (AL), gluteus maximus (GM), iliacus (IL), and tensor fasciae latae (TFL).

The setting of the computer used to train the model is configured as: GPU: NVIDIA GeForce RTX 3060 Ti; RAM: 16.0 GB.

### 2.2 Modified U-Net: Feature-Fusion-UNet/Attention-Feature-Fusion-UNet

In this study, we used U-Net (Ronneberger et al., 2015) and UNet++ as benchmarks for our segmentation task (Ronneberger et al., 2015; Dong et al., 2017). U-Net (Ronneberger et al., 2015; Zhu et al., 2021) is a 2D multi-layer Encoder-Decoder U-shaped neural network. The Encoder consists of convolutions and down sampling operations, enabling it to learn the features of input images and transmit them to the lower layers, generating a feature map, which is referred to as feature extraction. In the down sampling process, the receptive field gradually expands, resembling a compressed image, allowing for a larger perceived area per unit. Additionally, down sampling captures more low-frequency information from the image. The Decoder utilizes features to restore the original resolution of the feature map and perform pixel-level prediction. After each up-sampling operation, the output of the encoder at the corresponding layer is merged using skip connections. In U-Net, feature fusion is achieved by concatenating and merging features along the channel dimensions. UNet++ (Zhou et al., 2018) is a nested version of the U-Net architecture used for semantic segmentation, particularly in medical image analysis. It improves upon the U-Net by enhancing feature extraction and has shown superior performance in medical image segmentation tasks. It incorporates convolution layers on skip pathways to bridge the semantic gap and employs dense skip connections to enhance gradient flow, distinguishing it from the original U-Net.

Two modified U-Net networks were developed in this study. A Feature-Fusion-UNet (FFU) incorporated a fusion part into the basic U-Net model to improve the integration of semantic features from different layers. In FFU, the fourth, third, and second decoder layers’ feature mappings were using up-sampling operations to zoom in features of 8/4/2 times to maintain consistent sizes and then extracted by using the Atrous Spatial Pyramid Pooling (ASPP) (Chen et al., 2018) blocks to fuse the semantic information, which was subsequently concatenated with the output. Furthermore, to address potential issues such as gradient loss, a connection structure resembling the residual network was integrated into the top layer, facilitating improved gradient transfer.

**FIGURE 1:**
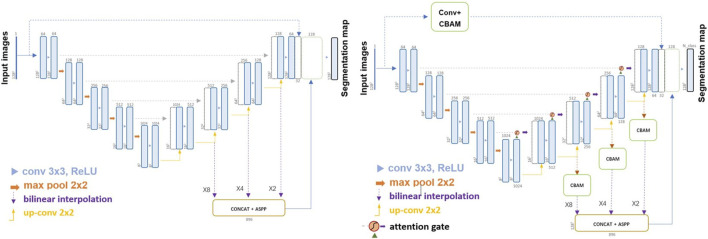
Structure of FFU (left) and AFFU (right).

Furthermore, the Attention-Feature-Fusion-UNet (AFFU) was proposed based on FFU to explore the employment of multiple attention mechanisms to achieve the desired segmentation accuracy. The AFFU incorporated attention gates into each individual layer (Oktay et al., 2018) and used CBAM block (Woo et al., 2018) to help compensate for any deficiencies in a single attention gate, and mitigated losses during resizing, enhancing the neural network’s receptive field using a pyramid pooling technique. The structure of FFU and AFFU is shown in Figure 1.

### 2.3 Data augmentation

The proposed data augmentation method was based on Statistical Shape Model (SSM) (Cates et al., 2007; Cates et al., 2017; Ravikumar et al., 2018; Clouthier et al., 2019).

Assuming that there are N samples with m correspondences on each contour, which can be seen as a 3×1 vector in shape space, $x_{k}$ represents a set of particles from k th shape, $x_{k}\in R^{3m},x_{k}=\left\{x_{k}^{1},x_{k}^{2},x_{k}^{3},\ldots ,x_{k}^{3m},\right\},k=\left\{1,2,\ldots ,N\right\}$ and let $Z=\left\{x_{1},x_{2},\ldots ,x_{N}\right\}$ , Z is a random variable of a shape in the shape space after aligning all shapes to the same coordinate system. Then the method consists in minimizing the energy function where H is an estimation of differential entropy.

$$Q=H\left(Z\right)-\sum_{N}H\left(x_{k}\right),$$ (1)

where the first term in Q minimization in (Eq. 1) targets a compact distribution of each individual’s sample in the shape space and the second term aims to increase the entropy of the correspondence distribution by achieving a more uniform distribution of points on each shape. The algorithm balances individual shape variation and overall shape similarity by minimizing both terms, allowing it to accurately model shape variations in the dataset.

The method was implemented using the publicly available software tool ShapeWorks (Cates et al., 2017). n different subject shape models of the same muscle were provided as input. The software then generated a mean shape model, from which n−1 modes were extracted using principal component analysis (PCA). Multiple deformations were produced based on the average shape model with different modes. Moreover, in order to generate new MR images associated to the newly generated muscle labels, a correspondence mapping between the original and new labels was calculated using the deformable registration algorithm Sheffield Image Registration Toolkit (ShIRT) (Barber and Hose, 2005; Henson et al., 2023a). By setting two parameters, the Nodal Spacing (NS) and the smoothing coefficient *λ*, ShIRT performed a non-linear deformable registration with high dimensional variability between input images or shapes. The two parameters of the deformable registration (NS and *λ*) were chosen from a previous sensitivity analysis equal to 5 voxels (5 mm) and 50, respectively (Henson et al., 2023b). The obtained mapping was applied to the original MR images using the same transformation matrix, resulting in a new set of segmented subjects without the need for manual segmentation.

**FIGURE 2:**
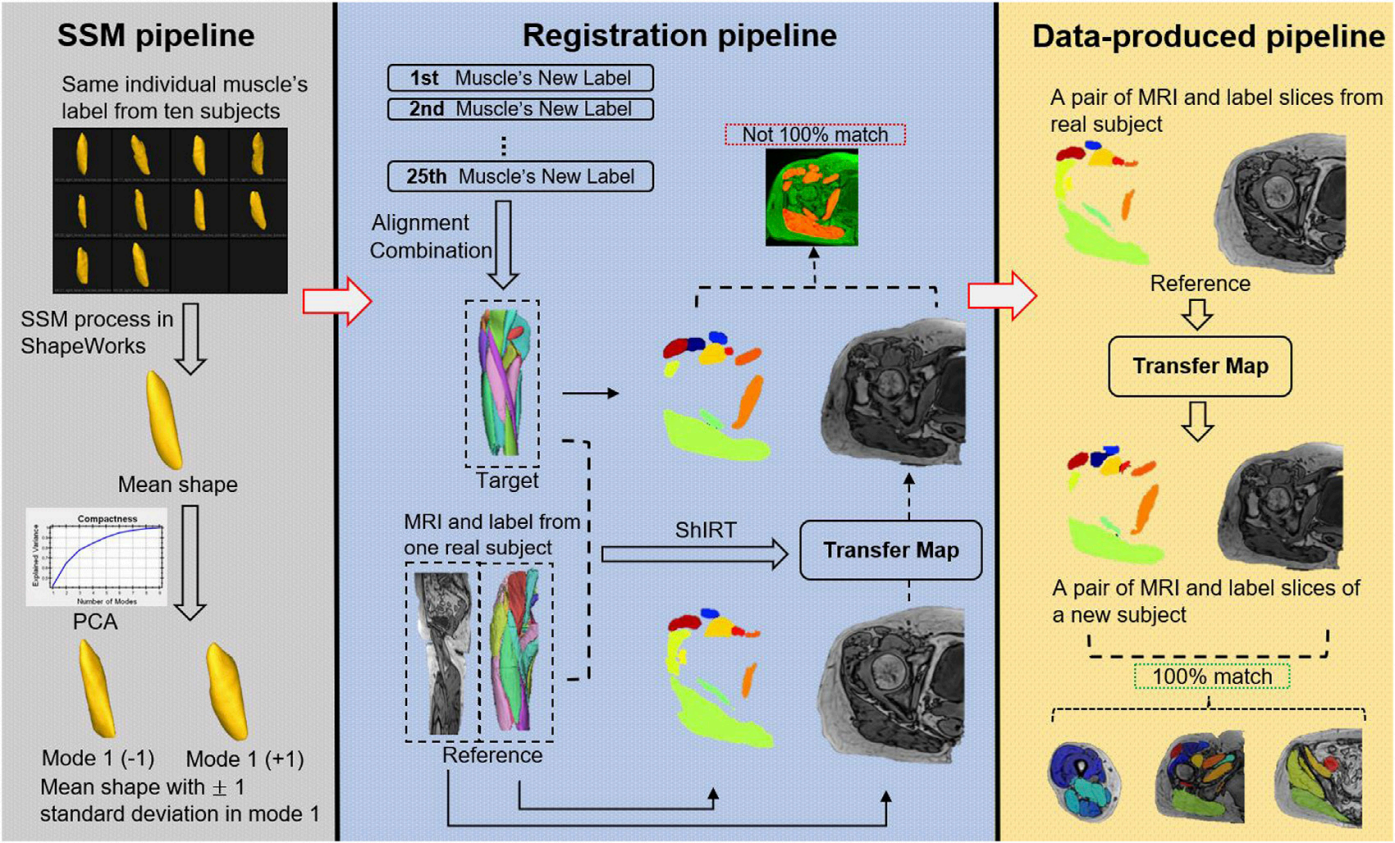
Data augmentation combined with Statistical Shape Model (SSM) process, registration, and data-produced pipelines. This figure illustrates the process for generating new subjects with matched labels without the need for manual image segmentation. The process is accomplished by using SSM and deformable image registration on ten original subjects’ MRI. The SSM pipeline (based on ShapeWorks software) inputs ten shapes of the same muscle from different subjects and applies an iterative optimization formula to generate corresponding points (default 1024 points) on all surfaces, calculates the mean shape, and uses PCA to extract nine principal features. New shapes are generated by adding or subtracting modes at different magnitudes from the mean shape. The registration pipeline involves obtaining new samples for all 25 muscles using SSM. A new muscle label (target) is formed by aligning the new samples with the original samples, and an original subject (reference) is selected. The MRI, label, and target’s label (mean shape) of the reference are inputted into ShIRT, which generates a mapping matrix that aligns the reference with the target labels. Using this map, the MR image of the original sample is transformed, resulting in an MR image that matches the target. However, due to image noise and the diversity of the target during registration, the transformed MR image may not perfectly overlap with the annotation generated by SSM (Barber et al., 2007), and therefore, cannot be directly used for subsequent model training. However, the transformation map generated by this pipeline can be preserved. The data-produced pipeline applies the mapping matrix generated by SSM to the label and MR image of the reference, resulting in fully matched image and label data (bottom right).

The pipelines of the proposed data-augmentation method are shown in Figure 2.

**FIGURE 3:**
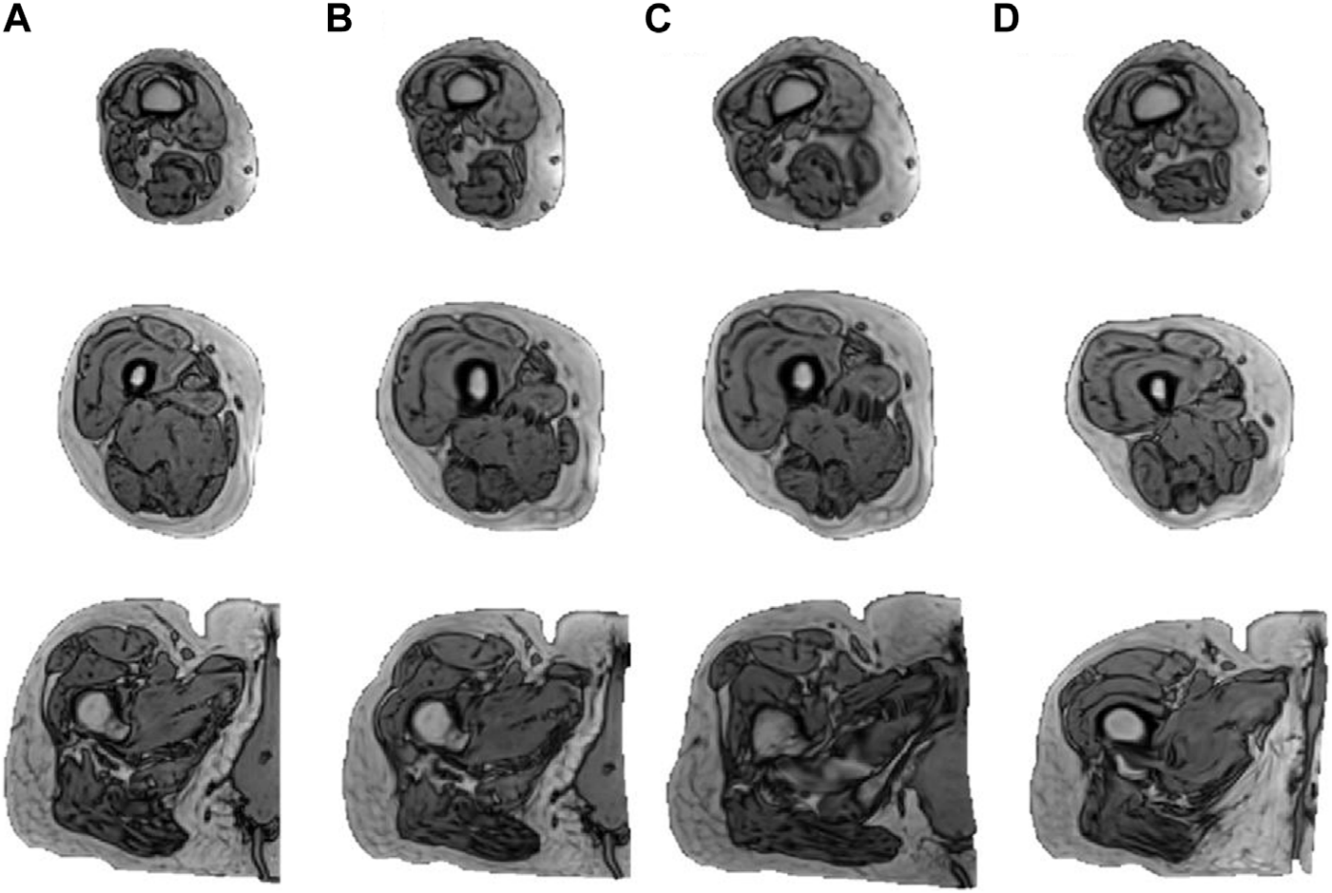
Augmentation results of one subject. The figure displays the outcomes derived from subjecting an individual to the augmentation process. Column **(A)** illustrates three slices of the original MRI (distal to proximal from top to bottom) from the right lower limb of one subject. **(B–D)** illustrate the results obtained by mapping the subject to the mean shape and mean shape ± 1 standard deviation mode in PCA, respectively.

Figure 3 reveals that the application of the ± 1 standard deviation mode to modify the mean shape yields noticeable alterations in volume and introduces heightened diversity in terms of texture within the generated new data.

### 2.4 Data pre-processing and training procedure

The dataset used in this study consisted of slices obtained from the right lower limb (knee to hip) of each subject. All images were cropped to a width of 256 pixels for input. During the model training process, random clipping was applied to further crop the input images to a width of 125 pixels to speed up training time and enhance the model’s robustness against noise.

To reduce the computational requirements, a batch size of 16 was chosen for the U-Net and UNet++, while a batch size of 10 was used for our models. The initial learning rate was set to 0.01 and decreased by a factor of 0.9 for each epoch. The model was trained for a total of 100 epochs, and the best weights on the validation set were retained for making predictions on the test set. The hyper-parameters were empirically tuned for best performance.

### 2.5 Experimental design

The experiment was split into three parts.

In the first part, four CNNs (U-Net, UNet++, FFU and AFFU) were trained and tested on PMW-1 data (11 subjects) using the leave-one-out approach. This approach was used to verify whether FFU and AFFU could improve the segmentation performance compared with the benchmark U-Net and UNet++ models.

The second part involved training on 10 subjects from PMW-1 and then utilizing the trained network to test on: the remaining 1 subject from PMW-1, 8 subjects from the PMW-2 dataset, and 10 subjects from the PMW-OB dataset. The objective of this part of the study was to evaluate the model’s generalization ability across different datasets.

For the third part, 10 subjects from the PMW-1 dataset used in the previous part were used as input to the augmentation pipeline, expanding the dataset to 37 subjects in total, based on the mean shape of the muscles and including a variability of ±1 standard deviation associated to the PCA mode that describes best the variability in the dataset (mode 1). The augmented dataset was then used to re-train the model weights obtained from the second part of the study. This part of the study aimed to investigate whether training with the augmented dataset improved the segmentation performance of each model on the testing data (1xPMW-1, 8xPMW-2, 10xPMW-OB).

### 2.6 Evaluation metrics

For each part of the study three metrics were used: Dice Similarity Coefficient (DSC), Relative Volume Error (RVE), and Hausdorff distance (HD) (Dong et al., 2017; Zhu et al., 2021; Henson et al., 2023b). These metrics provide complementary insights into the performance of the segmentation models and help assess their accuracy, volume similarity, and the degree of local errors.

The Dice Similarity Coefficient (DSC) was calculated by using Eq. 2, which shows the similarity between reference shapes (R) and predicted shapes (P).

$$DSC=\frac{2\left|R\cap P\right|}{\left|R\right|+\left|P\right|}$$ (2)

where R is the set of voxels in the reference labels from manual work and P is from the model’s prediction results. A DSC equal to 1 indicates a perfect match between the segmentation result and the ground truth, while a value of 0 represents no overlap.

Relative Volume Error (RVE) was utilized to assess the similarity between the segmentation results and the ground truth in terms of total volume of the individual muscle. RVE was defined as in Eq. 3

$$RVE=\frac{V_{ref}-V_{pred}}{V_{ref}}\times 100\%$$ (3)

where $V_{ref}$ and $V_{pred}$ are the volume of reference and prediction cohort respectively. For the convenience of display, 1-RVE was used as measurement of accuracy.

The Hausdorff distance (HD) is a metric that measures the maximum distance between the reference and predicted external surfaces. It provides information about the extent of local errors in the model’s prediction. HD was calculated as in Eq. 4

$$HD\left(R,P\right)=\max\left\{d\left(R,p\right),d\left(r,P\right)\right\}$$ (4)

where r is the voxel on surface of R and p is the voxel on surface of P , d is a function to find the minimum distance between r or p and the nearest point within the cohort P or R .

### 2.7 Statistics

When comparing two different models the difference between same individual muscle metrics were tested for normality using a Kolmogorov-Smirnov test, concluding that the difference between the paired values were not normally distributed. Therefore, a Wilcoxon signed-rank test was utilized. The paired values of each model’s DSC, 1-RVE and HD metrics were compared across the four models. A significance level *α* = 0.05 was considered.

## 3 Results

### 3.1 Performance of the models on the PMW-1 cohort

**FIGURE 4:**
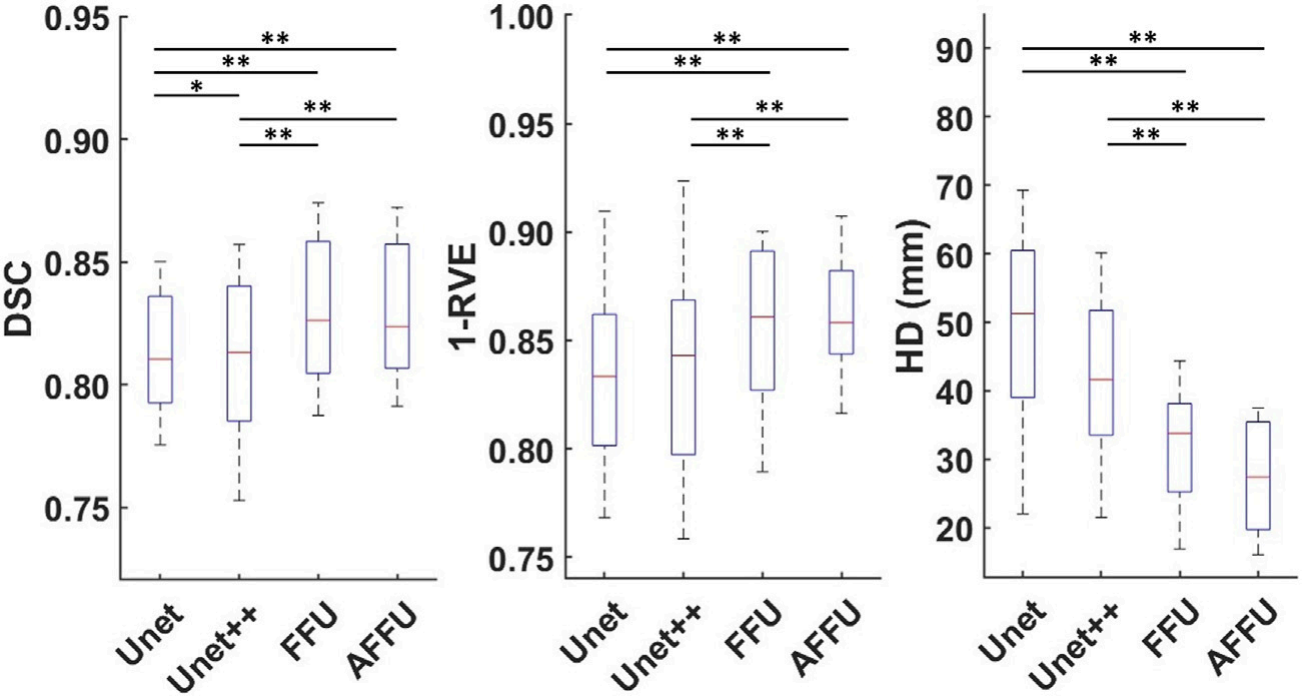
Performance of each model established using cross-validation. Box plots for the DSC (left), 1-RVE (middle) and HD (right, lower scores indicate better performance) for each model tested on the PMW-1 cohort (16 mean values of each individual muscle calculated from 176 predicted labels) are shown (* indicates *p* < 0.05, ** indicates *p* < 0.01).

In total, each model predicted 176 labels representing one lower limb muscle (11 subjects with 16 muscle labels each) through cross-validation. Supplementary Table S1 presents the accuracy of each model to predict the three considered metrics, DSC, 1-RVE and HD. Significant differences between the accuracy of the models and the accuracy of the U-Net or the UNet++ are reported in Figure 4.

**FIGURE 5:**
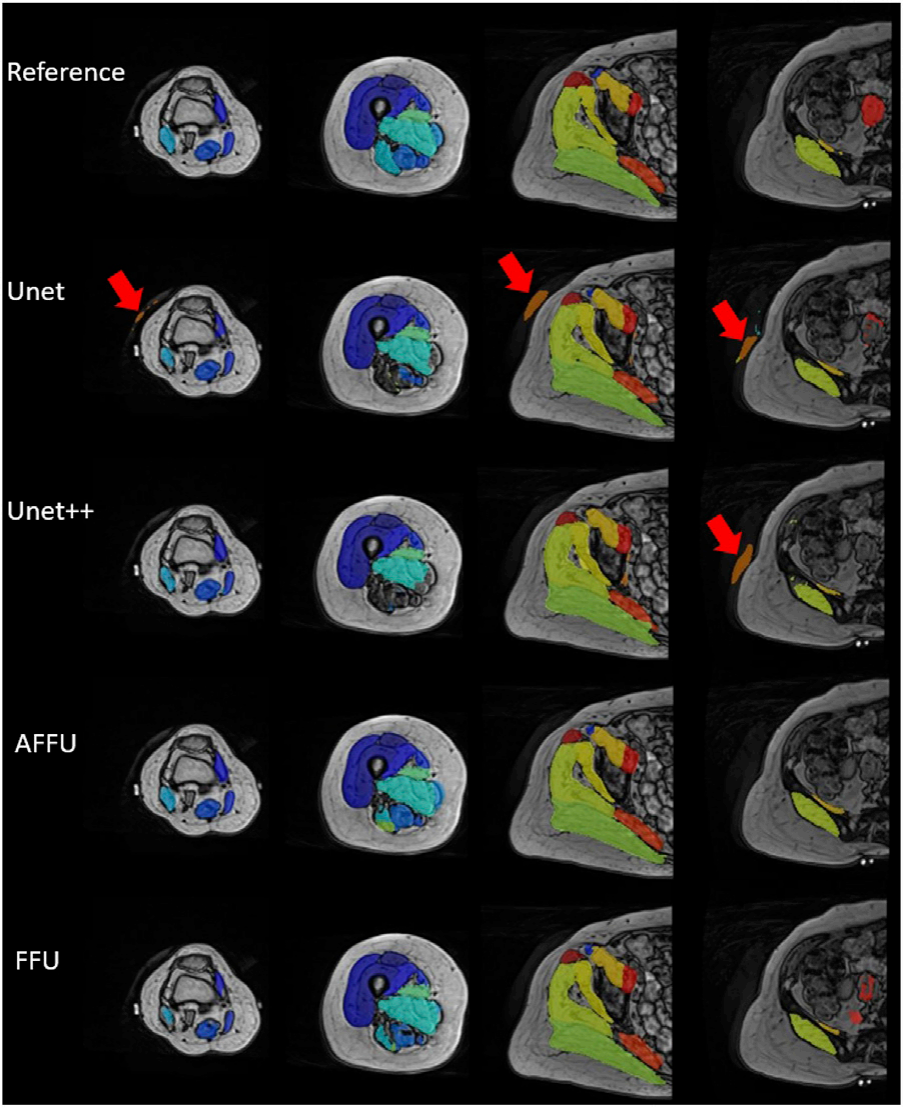
Segmentation visualization of muscles of one subject from prediction of each model and manual reference. The first row represents the gold standard generated by manual muscle segmentation by experienced operators. In the second and third rows, the performance of U-Net and UNet++ is depicted, wherein some local errors (red arrows) around the contour compared to the reference are noticeable, along with a few mis-segmented points. The fourth and fifth rows present the results obtained from AFFU and FFU, which exhibit generally superior segmentation compared to U-Net and UNet++.

Overall, the AFFU was found to have the best performance among all four, producing the best average DSC (0.828), RVE (0.859) and HD (29.9 mm) across 16 muscles. FFU was the second-best model with DSC (0.826), RVE (0.853) and HD (32.4 mm). On the test set, both models outperformed U-Net (and UNet++) with the following average improvements: 2.2% (2.1%) in DSC, 3.3% (2.6%) in 1-RVE, 40.1% (33.1%) in HD for AFFU; 2.0% (1.8%) in DSC, 2.5% (1.9%) in 1-RVE, 35.1% (27.5%) in HD for FFU. The mean metrics value of different muscles for each model are shown in the (Supplementary Table S2). Figure 5 shows the segmentation visualization results of some muscles, where it could be observed that the results obtained from AFFU and FFU, exhibit generally superior segmentation compared to U-Net and UNet++.

### 3.2 Performance of the models on different cohorts after training on the PMW-1 cohort

**FIGURE 6:**
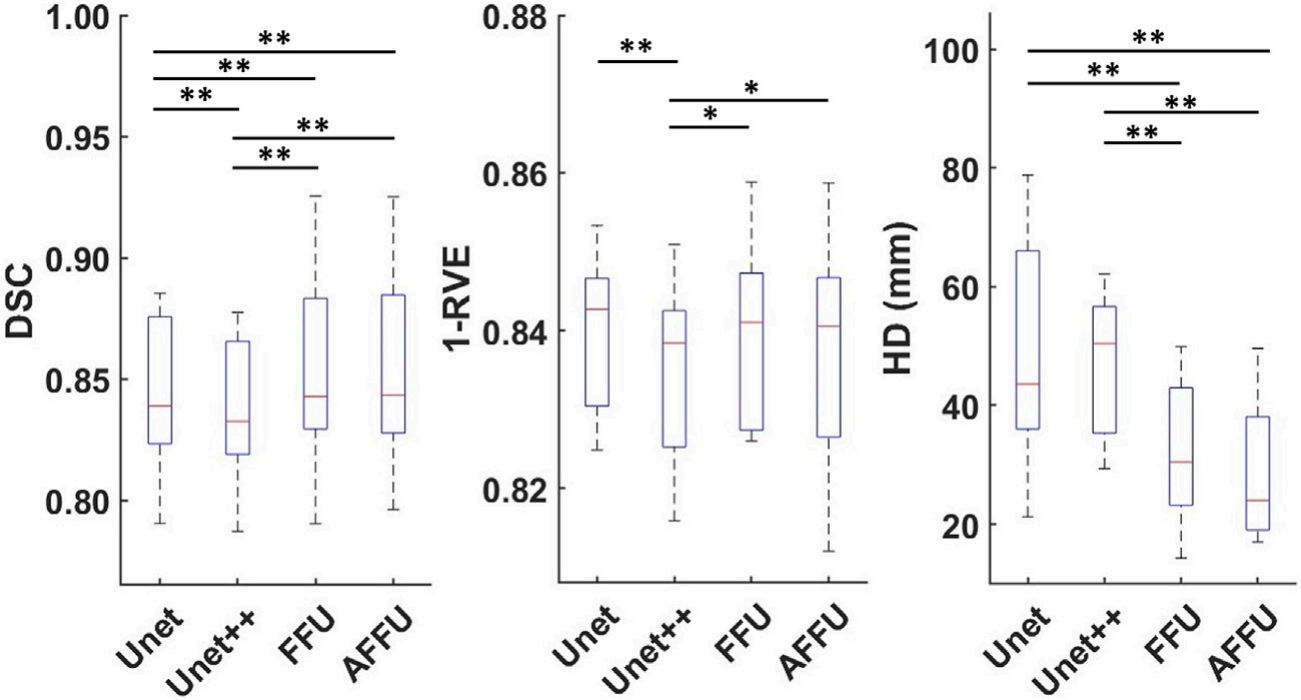
Performance of each model on all 19 test subjects. For each model and each metric, 16 mean values calculated from 304 predicted labels (16 labels for each of the 19 subjects) are shown (* indicates *p* < 0.05, ** indicates *p* < 0.01).

In this section, three datasets (i.e., PMW-1, PMW-2, and PMW-OB cohorts), were used to evaluate the performance of the models after having trained the models on the data from the PMW-1 cohort. Supplementary Table S3 and Figure 6 provide an overview of the average performance of each model, pooling together the results from different subsets (1xPMW-1, 8xPMW-2, 10xPMW-OB).

The AFFU and FFU models were more accurate in segmenting the muscles compared to the U-Net (0.59% and 0.59% higher DSC, respectively; 39% and 27% lower HD, respectively) and the UNet++ (1.31% and 1.31% higher DSC, respectively; 1.13% and 1.13% higher 1-RVE, respectively; 42% and 31% lower HD, respectively). The AFFU also showed a significant improvement compared to FFU on HD (15.9%, *p* = 0.010). The full results for individual muscles and each model are shown in Supplementary Table S4.

The performance of each model for segmenting the muscles of the 8 subjects from the PMW-2 cohort and the 10 subjects from the PMW-OB cohort are reported in Supplementary Table S5. In terms of (1-RVE), there were no significant differences in accuracy of the models. Each model achieved high (1-RVE) values in the range 0.87–0.92, indicating accurate volume estimation. For DSC, AFFU demonstrated a gain of approximately 0.6% over U-Net in both groups (*p* = 0.008 for PMW-2, *p* < 0.001 for PMW-OB). FFU showed a significant improvement compared to U-Net for the PMW-2 (+0.72%, *p* = 0.006) and PMW-OB (0.47%, *p* = 0.006) cohorts. For HD, both AFFU and FFU consistently exhibited smaller local errors compared to U-Net (22.0%–42.4%, *p* < 0.001). On average, they achieved a reduction of approximately 20 mm in local distance, indicating improved localization accuracy.

All muscle segmentation results are reported in Supplementary Tables S6, S7.

### 3.3 Effect of augmentation data

**FIGURE 7:**
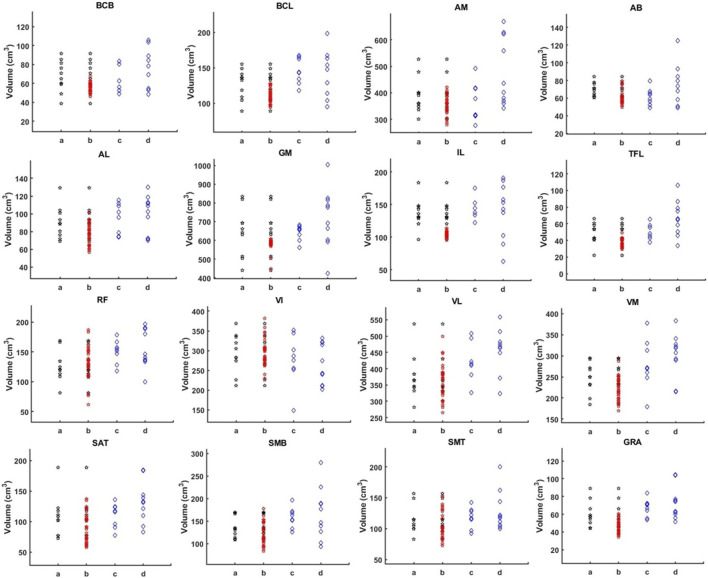
Muscle volume distribution. The figure illustrates the volume distribution of 16 muscles in four sets: 10 PMW-1 training samples (column a), training samples with added augmentation data (column b), PMW-2 (column c), and PMW-OB (column d) cohorts. In each column b, the red dots represent the distribution of the additional segmented subjects, while the black dots represent the values from the original scans.

The muscle volume distribution after the addition of the augmented data is reported in Figure 7, showing that the augmented data fills some gaps in the original data distribution. If tested on the all subjects pooled together, small significant improvements in DSC were observed by using the augmented training dataset (*p* < 0.001, +0.6% for AFFU; *p* < 0.001, +1.5% for U-Net, Table 1). However, when considering 1-RVE and HD, the performance remained almost unchanged (very small 0.9% improvement in RVE was observed for U-Net with the augmented dataset). The full results can be found in Supplementary Table S8.

**TABLE 1:** Model comparison under three metrics with/without augmentation data on all test subjects pooled together.

		Without aug.	With aug.	p	Difference [%]
DSC Mean ± SD [%]	U-Net	0.843 ± 0.062	0.856 ± 0.054	**<0.001**	1.54
	AFFU	0.848 ± 0.058	0.853 ± 0.055	**<0.001**	0.59
RVE Mean ± SD [%]	U-Net	0.893 ± 0.097	0.901 ± 0.091	**<0.001**	0.90
	AFFU	0.894 ± 0.096	0.897 ± 0.094	0.286	NS
HD Mean ± SD [mm]	U-Net	49.1 ± 52.8	43.5 ± 51.3	**<0.001**	11.40
	AFFU	30.2 ± 38.7	31.3 ± 43.4	**0.002**	−3.64

That the bold values indicates the *p*-value is significant.

U-Net demonstrated a noticeable increase in the DSC for all three cohorts (PMW-1: 1.2%, *p* = 0.006; PMW-2:2.2%, *p* < 0.001; PMW-OB:1.1%, *p* < 0.001, Table 2). On the other hand, AFFU shows a small significant increase in the DSC only for PMW-2 (1.1%, *p* < 0.001, Table 2). Significant improvements in RVE, and HD for the PMW-2 cohort were observed (+1.93% for U-Net and +1.6% for AFFU in RVE, and +19.8% for U-Net in HD, Table 2). In contrast, in PMW-OB, only the DSC metric showed significant improvement, while RVE and HD remained relatively unchanged. The results obtained for single muscles are reported in Supplementary Tables S9–11.

**TABLE 2:** Comparison of models across three metrics for different cohorts trained with augmentation data. The significant *p*_value should be shown in bold.

PMW-1					
		Without aug.	With aug.	p	Difference [%]
DSC (Mean ± SD [%])	U-Net	0.824 ± 0.051	0.834 ± 0.047	**0.006**	1.21
	AFFU	0.833 ± 0.056	0.836 ± 0.050	0.796	NS
RVE (Mean ± SD [%])	U-Net	0.786 ± 0.145	0.780 ± 0.156	0.918	NS
	AFFU	0.801 ± 0.104	0.806 ± 0.125	0.717	NS
HD Mean ± SD [mm]	U-Net	27.2 ± 12.1	22.4 ± 9.0	**0.020**	17.65
	AFFU	19.7 ± 7.5	17.8 ± 7.0	**0.011**	9.64
PMW-2					
DSC (Mean ± SD [%])	U-Net	0.828 ± 0.073	0.846 ± 0.061	**<0.001**	2.17
	AFFU	0.833 ± 0.065	0.842 ± 0.060	**<0.001**	1.08
RVE (Mean ± SD [%])	U-Net	0.880 ± 0.105	0.897 ± 0.094	**<0.001**	1.93
	AFFU	0.873 ± 0.105	0.887 ± 0.100	**<0.001**	1.60
HD Mean ± SD [mm]	U-Net	45.0 ± 47.6	36.1 ± 39.9	**<0.001**	19.8
	AFFU	25.9 ± 27.9	27.0 ± 30.1	0.065	NS
PMW-OB					
DSC (Mean ± SD [%])	U-Net	0.857 ± 0.049	0.866 ± 0.046	**<0.001**	1.05
	AFFU	0.862 ± 0.048	0.864 ± 0.049	0.097	NS
RVE (Mean ± SD [%])	U-Net	0.914 ± 0.072	0.916 ± 0.069	0.472	NS
	AFFU	0.919 ± 0.076	0.913 ± 0.079	0.067	NS
HD Mean ± SD [mm]	U-Net	54.5 ± 58.4	51.6 ± 59.9	0.380	NS
	AFFU	34.8 ± 46.8	36.2 ± 52.9	0.071	NS

**FIGURE 8:**
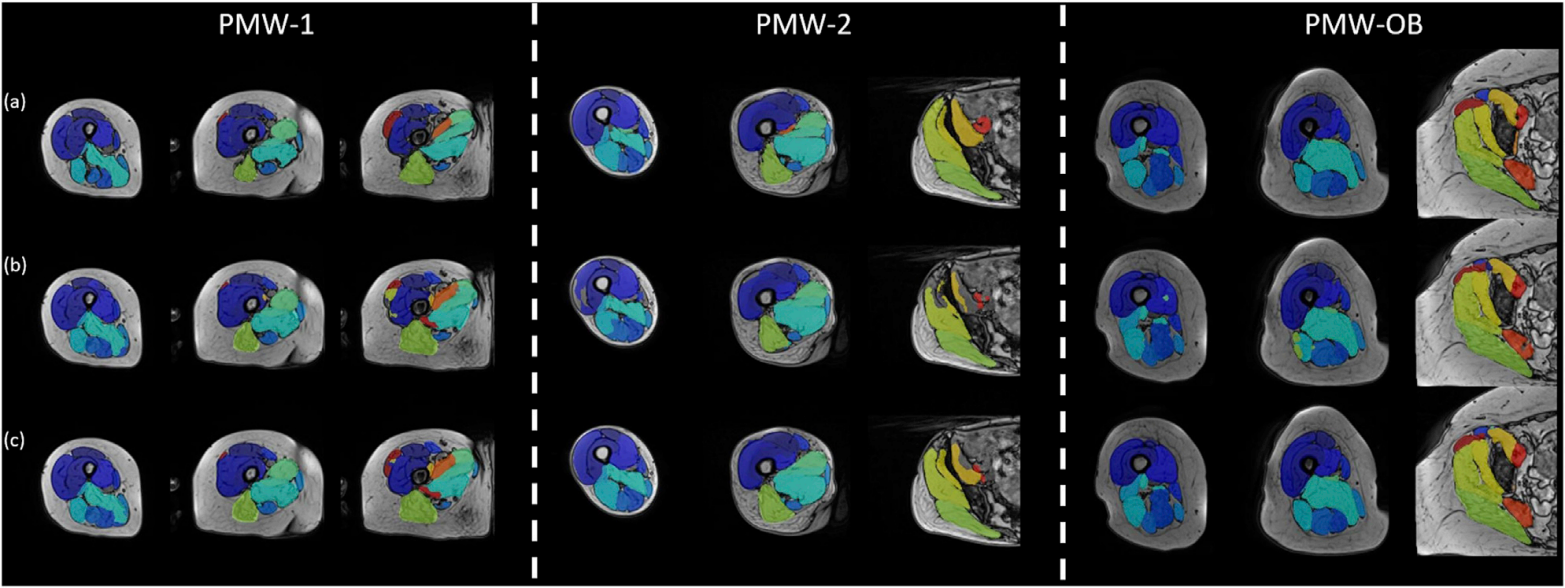
Segmentation performance visualization of U-Net trained without/with augmentation data. The figure exhibits the segmentation performance of U-Net on a subject chosen from each of the three cohorts (PMW-1/2/OB). (a) displays the gold standard manual segmentation, while (b) and (c) present the results for the U-Net model trained without or with augmented data, respectively.

Augmenting the training data has enabled U-Net to learn additional sample features, thereby enhancing its performance and decreasing the likelihood of segmentation errors, for example by effectively filling the muscle volume that were previously not well identified (Figure 8).

### 3.4 Analysis for individual muscles

The PMW-OB cohort had generally higher mean muscle volumes for the muscles with volume in the high range (VM, VL, AM, GM) compared to the other two cohorts.

**FIGURE 9:**
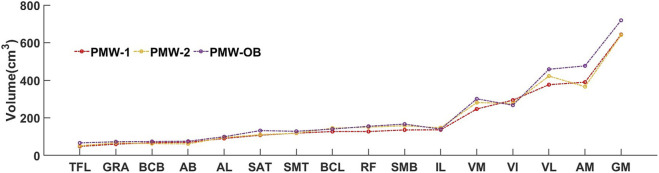
Individual muscle volume comparison among PMW-1/2/OB cohorts.

The muscles were ranked in order of volume for the PMW-1 cohort (mean volume: 189.9 ± 162.5 cm^3^). Compared with PMW-1 and PMW-2 (197.2 ± 162.8 cm^3^) cohorts, some muscles such as VL, AM and GM in the PMW-OB (217.1 ± 185.9 cm^3^) cohort showed higher values (Figure 9).

**FIGURE 10:**
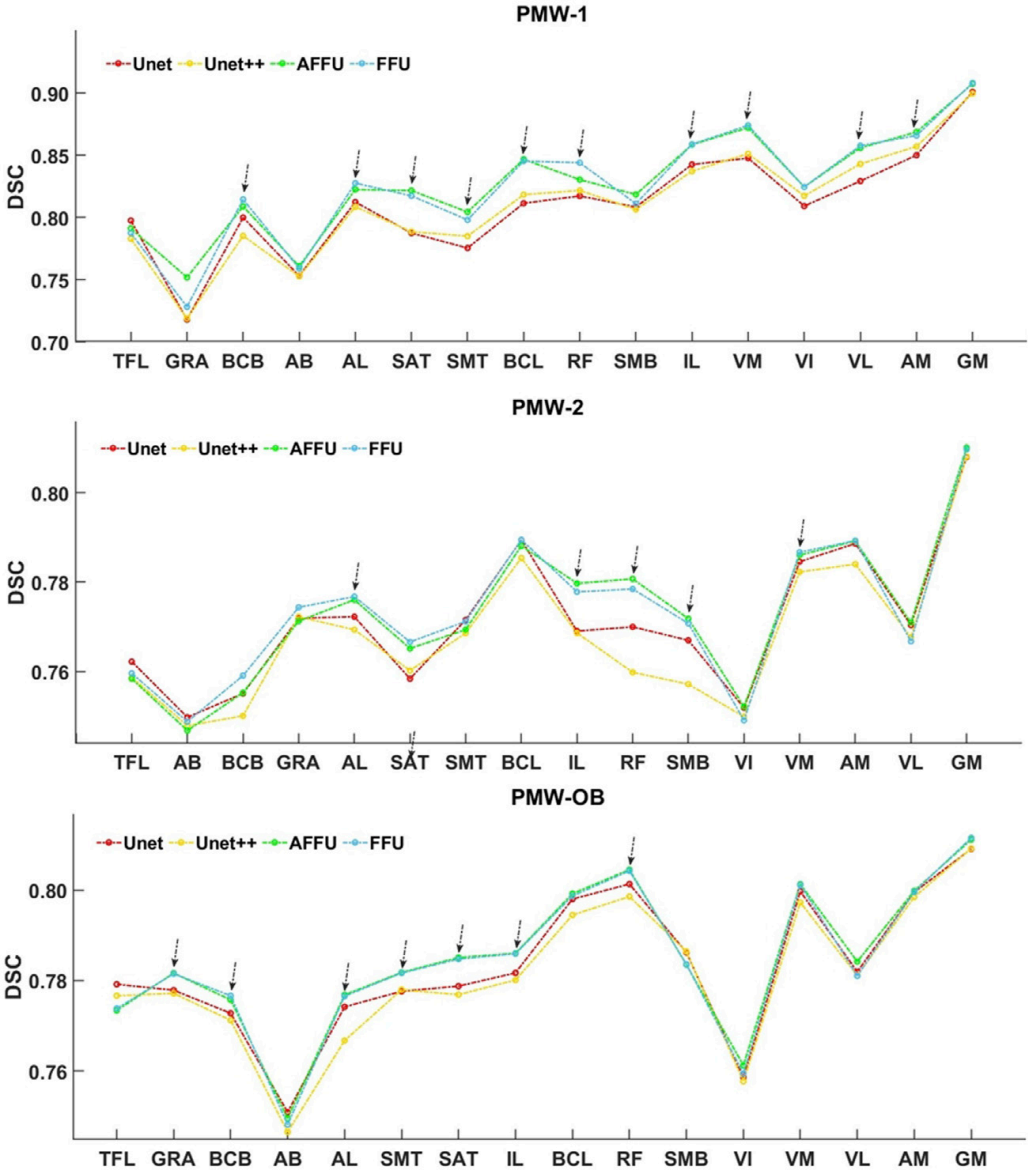
Relationship between muscle volume and DSC index in women cohorts. Muscle volume and DSC for the three different cohorts of subjects. The muscles were arranged in ascending order according to the mean muscle volume of each specific cohort, and each point in the figure represents the average DSC value of the muscle across the subjects.

Overall, a trend of increasing DSC for all models was observed as muscle volume increased (Figure 10). In particular, for the PMW-1 and PMW-2 cohorts, muscles with volume in the middle range were better segmented by AFFU and FFU compared to U-Net (improvement of 0.72%–4.71% (PMW-1), 0.17%–3.23% (PMW-2) for AFFU and 0.78%–4.19% (PMW-1),0.18%–2.66% (PMW-2) for FFU, for most muscles with volume in the middle or middle-high range (arrows in Figure 10). Similar trends were found for the PMW-OB cohort, with AFFU that led to better predictions than U-Net for most muscles with volume in the middle-low range (improvement of 0.33%–1.87%). For the PMW-1 cohort a not-significant trend of increased accuracy in DSC for larger muscles was found (Figure 10).

**FIGURE 11:**
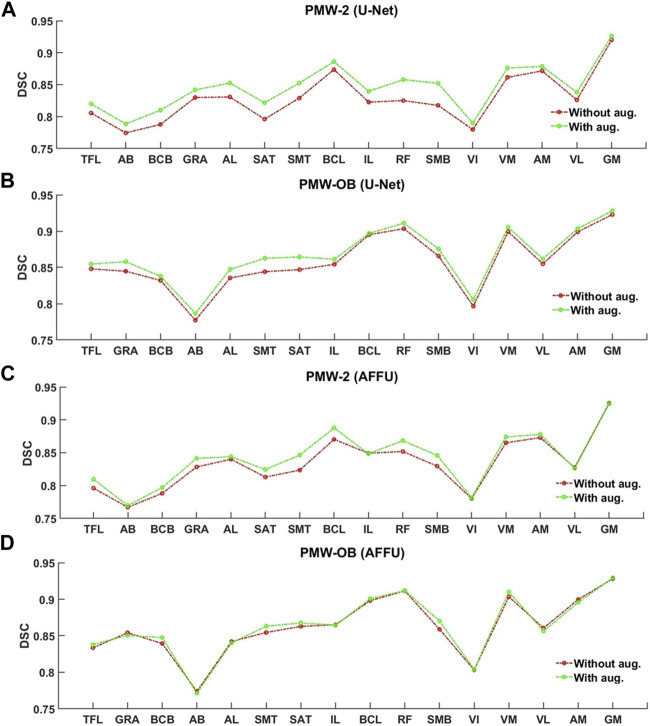
Per-muscle evaluation trained with/without augmentation of U-Net **(A,B)** and AFFU **(C,D)**. The plots display the DSCs before and after data augmentation.

Data augmentation affected similarly the segmentation of the individual muscles, with main gains for the U-Net and AFFU model applied to the PMW-2 cohort (Figure 11).

## 4 Discussion

This study aimed to evaluate the performance of different DL models to automatically segment individual muscles of the lower limbs from the MRI images of different cohorts of women. The new models proposed in this study, that include the fusion part (FFU) and multi-added attention mechanism (AFFU), were found to be significantly more accurate than the standard U-Net and UNet++ models. The proposed data augmentation method also enhanced the segmentation accuracy of the models trained on small datasets.

AFFU achieved average DSC values of 0.83–0.85 depending on the training dataset, providing a reasonable automatic segmentation of most muscles even if trained on different cohorts of women. In this study, models were trained on same dataset and tested on different cohorts. Higher accuracy was found for previous automatic segmentation tools trained and tested on young cohorts (DSC of 0.85 and 0.91 in (Ni et al., 2019; Zhu et al., 2021), respectively). The difference may be due to differences between this study and previous studies: fewer training datasets were used in this study (11 subjects versus 20–60 subjects (Ni et al., 2019; Zhu et al., 2021)); more complex image texture was processed for the tested cohorts including post-menopausal women and obese post-menopausal women compared to the younger healthy subjects in those studies; training was performed on the PMW-1 cohort and then tested on the other two cohorts, evaluating the possible application of the models if not trained on the same cohort. While the accuracy of the current approach may not be enough for applying it directly in clinical settings, it has a lot of potential for application in a research environment. For example, it could be used to automatically identify the individual muscles properties (e.g. muscle volume) used for the personalized biomechanical assessment of the risk of femoral fracture (Altai et al., 2021).

AFFU and FFU models showed better performance than U-Net and UNet++ models when tested on different cohorts. This effect indicates that feature fusion part and attention mechanisms could learn more features than U-Net in a small sample size (n∼10) and the fusion of features from layers 2/3/4 into U-Net could enhance its learning capability. Moreover, the addition of attention mechanisms led to more robust performance across different samples. This improvement may be due to the fact that the attention mechanism allows the model to notice more general features, rather than information that is specific to post-menopausal women. The results also indicate that the proposed models are more resistant to noise interference compared to the standard models. Although the differences in DSC may not be significant for some results, the HD, that is associated with local errors, showed a significant improvement of approximately 15–25 mm compared to the benchmark. As the muscle MR scans approaches the hip and its image texture becomes more complex, there is a discernible rise in the occurrence of mis-segmentation areas within the U-Net predictions. These areas can be attributed to noise interference, underlining the need for further investigation into the factors influencing the segmentation accuracy. As can be observed in Figures 8, 9, there were a few fat infiltrations in the muscles of the PMW-OB cohort, which were generally larger than those of the other two cohorts for the larger muscles. Assuming that the neural network has the same ability to identify the target region during segmentation, the impact of noise on parameters such as DSC and RVE is less obvious in numerical values when working on larger muscles. This shows that on some fat or edge treatments, the model still does not learn relevant features from the PMW-1 cohort to the PMW-OB cohort. Even so, the results of the model on PMW-OB (U-Net: DSC = 0.857 ± 0.049; AFFU: DSC = 0.862 ± 0.048) cohort are comparable compared to segmentation results on younger subjects in the literature (Ni et al., 2019; Zhu et al., 2021). U-Net and AFFU exhibited generally superior performance for the same muscles in the PMW-OB cohort. This indicates that a higher proportion of fat might have a larger impact on noise in segmentation, leading to larger local errors (higher HD). However, this should be confirmed with further analysis to determine if the segmentation accuracy is influenced by the training with data from different cohorts.

When training data augmentation based on the PMW-1 cohort was used, improvements were found mainly for U-Net tested on the PMW-1 cohort, as expected. The improvement of the AFFU and FFU models was not particularly evident when tested on the PMW-2 and PMW-OB cohorts. This effect could be due to the simpler structure of U-Net, which lacks attention mechanisms or other complex structures, leading to its higher sensitivity to the quantity of training data. In contrast, the model with attention mechanisms can learn more features and information with relatively fewer subjects. Deep learning is a supervised learning approach, with the amount of data often determining the depth of the model’s learning. The U-Net and AFFU models achieved a similar level of accuracy in segmenting the muscles after training with augmentation data, indicating that the models learned as much relevant information as possible from the dataset. Moreover, considering that the training dataset was based only on the data from the PMW-1 cohort, the improvement in accuracy for the PMW-2 and PMW-OB cohorts is probably due to the inclusion in the enlarged dataset of muscle features also beneficial for the other cohorts. Nevertheless, it remains to be investigated what would happen if the augmentation would be based also on the PMW-2 and PMW-OB cohorts, which will be tested in future work.

The DSC performance was positively correlated with muscle volume for most models and muscles (except AB and VI). This trend shows that the automatic segmentation performance of the model is generally better for larger muscles. However, more variability was found for the testing cohorts PMW-2 and PMW-OB. This result is important, especially if the segmentation of the models is used to assess the function of large muscles due to musculoskeletal diseases (Galbusera et al., 2020), or for creating biomechanical applications of personalized multi-body dynamics models (Graffy et al., 2019), applications for which it is more important to assess the properties of large muscles. Nevertheless, for improving the applicability of the models, they should be better optimized for automatically segmenting smaller muscles, such as TFL and GRA, where there is more room for improvement. This trend is not reflected in the literature (Ni et al., 2019), due to the fact that there is less difference between the muscles of the young athlete sample compared to the older women sample, and with higher image definition. Nevertheless, the variability is also found generally higher in small volume muscles like quadratus femoris (DSC = 0.81 ± 0.057) or muscles with irregular shapes in (Ni et al., 2019).

The technique developed in this study lies in the feature fusion and attention fusion, which has been demonstrated to be highly beneficial for muscle segmentation of post-menopausal women compared to the classical U-Net (Ronneberger et al., 2015). Moreover, it is noteworthy that the proposed models are generic and modular as such it can be easily applied to natural/medical image segmentation, classification, as well as regression problems.

AFFU/FFU spent around 10 h in the training period, but less than 0.5 s per MR image on the test period. When using a well-trained model to make a new subject prediction, it takes less to segment a subject compared to the manual segmentation (Montefiori et al., 2020; Henson et al., 2023a). In this study it was decided to train and test the models by using a standard research workstation, in order to increase the applicability of the model and do not require to run it by using high performance computing (HPC) cluster. Nevertheless, the training and running time can be reduced by using an HPC cluster in the future.

This study is affected by some limitations. Firstly, it is evident that despite using data augmentation techniques, the limited quantity of data from real subjects still significantly affects model training. Although no overfitting was observed during the training phase by monitoring the loss function’s decline, training with a larger number of independent real subjects should be further considered in future studies. Moreover, considering the potential bias between different cohorts, more training data from different cohorts of subjects could be added to enrich the features that can be learned by the model and improve therefore its accuracy. Furthermore, a post-processing step (Lafferty et al., 2001) has the potential of further improving the accuracy of the models. Different post-processing approaches will be tested in a future study. A second limitation relates to the proposed novel data augmentation approach, which uses only the feature with the highest variation from the PCA analysis. In future studies, incorporating more features and extending the variability of those may lead to the creation of realistic subjects across a wide range of volumes and shapes, with the potential of improving the segmentation accuracy of the models also for different cohorts of subjects. Lastly, after visualizing the model’s segmentation results, it became apparent that some muscles exhibited noticeable segmentation errors when observed in 3D. These segmentation errors could potentially be mitigated through post-processing approaches. Since muscles generally exhibit specific geometric shapes, leveraging geometric models in post-processing might alleviate some limitations inherent in the model itself.

## 5 Conclusion

This study has shown that using multi attention mechanisms and fusion structure improved the automatic segmentation of muscles from MR images from different cohorts of post-menopausal women, compared to standard U-Net models. Moreover, it has been shown that these models are less affected by the number of used training datasets, compared to the U-Net model, which benefited from the augmentation of the training dataset.

In order to optimizing this approach for clinical research, further analyses should focus on improving the models for automatically segmenting smaller muscles and testing the applicability of the models when trained with larger number of subjects from different cohorts.

## Data Availability

The original contributions presented in the study are included in the article/Supplementary Material, further inquiries can be directed to the corresponding author.

## References

[B1] Adams R. Bischof L. (1994). Seeded region growing. IEEE Trans. Pattern Analysis Mach. Intell. 16 (6), 641–647. 10.1109/34.295913

[B2] Altai Z. Montefiori E. Van Veen B. Paggiosi M. A. McCloskey E. V. Viceconti M. (2021). Femoral neck strain prediction during level walking using a combined musculoskeletal and finite element model approach. PLOS ONE 16 (2), e0245121. 10.1371/journal.pone.0245121 33524024 PMC7850486

[B3] Barber Dc Hose Dr (2005). Automatic segmentation of medical images using image registration: diagnostic and simulation applications. J. Med. Eng. Technol. 29 (2), 53–63. 10.1080/03091900412331289889 15804853

[B4] Barber D. C. Oubel E. Frangi A. F. Hose D. R. (2007). Efficient computational fluid dynamics mesh generation by image registration. Med. Image Anal. 11 (6), 648–662. 10.1016/j.media.2007.06.011 17702641

[B5] Cates J. Elhabian S. Whitaker R. (2017). “Shapeworks: particle-based shape correspondence and visualization software,” in Statistical shape and deformation analysis (London: Academic Press).

[B6] Cates J. Thomas Fletcher P. Styner M. Shenton M. Whitaker R. (2007). Shape modeling and analysis with entropy-based particle systems. Inf. Process. Med. Imaging 4584, 333–345. 10.1007/978-3-540-73273-0_28 PMC2768473 17633711

[B7] Chen L.-C. George P. Kokkinos I. Murphy K. Alan L. (2018). DeepLab: semantic image segmentation with deep convolutional nets, atrous convolution, and fully connected CRFs. IEEE Trans. Pattern Analysis Mach. Intell. 40 (4), 834–848. 10.1109/TPAMI.2017.2699184 28463186

[B8] Clouthier A. L. Smith C. R. Vignos M. F. Thelen D. G. Deluzio K. J. Rainbow M. J. (2019). The effect of articular geometry features identified using statistical shape modelling on knee Biomechanics. Med. Eng. Phys. 66, 47–55. 10.1016/j.medengphy.2019.02.009 30850334 PMC6529205

[B9] Cruz-Jentoft S. Alfonso J. Baeyens J. P. Bauer J. M. Boirie Y. Cederholm T. (2010). Sarcopenia: European consensus on definition and diagnosis. Age Ageing 39 (4), 412–423. 10.1093/ageing/afq034 20392703 PMC2886201

[B10] Davico G. Bottin F. Di Martino A. Castafaro V. Baruffaldi F. Faldini C. (2022). Intra-operator repeatability of manual segmentations of the hip muscles on clinical magnetic resonance images. J. Digital Imaging 36 (1), 143–152. 10.1007/s10278-022-00700-0 PMC9984589 36219348

[B11] Dong H. Yang G. Liu F. Mo Y. Guo Y. (2017). Automatic brain tumor detection and segmentation using U-net based fully convolutional networks. Commun. Comput. Inf. Sci. 723, 506–517. 10.1007/978-3-319-60964-5_44

[B12] D’Souza A. Bolsterlee B. Lancaster A. Herbert R. D. (2019). Muscle architecture in children with cerebral palsy and ankle contractures: an investigation using diffusion tensor imaging. Clin. Biomech. 68, 205–211. 10.1016/j.clinbiomech.2019.06.013 31255994

[B13] Galbusera F. Cina A. Panico M. Albano D. Messina C. (2020). Image-based biomechanical models of the musculoskeletal system. Eur. Radiol. Exp. 4 (1), 49. 10.1186/s41747-020-00172-3 32789547 PMC7423821

[B14] Graffy P. M. Liu J. Pickhardt P. J. Burns J. E. Yao J. Summers R. M. (2019). Deep learning-based muscle segmentation and quantification at abdominal CT: application to a longitudinal adult screening cohort for sarcopenia assessment. Br. J. Radiology 92 (1100), 20190327. 10.1259/bjr.20190327 PMC6724622 31199670

[B15] Helen R. Kamaraj N. Selvi K. Raja Raman V. (2011). “Segmentation of pulmonary parenchyma in CT lung images based on 2D otsu optimized by PSO,” in 2011 International Conference on Emerging Trends in Electrical and Computer Technology, Nagercoil, India, 23-24 March 2011 (IEEE). 10.1109/ICETECT.2011.5760176

[B16] Henson W. H. Lin X. Lin Z. Guo L. Mazzá C. Dall’Ara E. (2023b). Automatic segmentation of lower limb muscles from MR images of post-menopausal women based on deep learning and data augmentation. arXiv. 10.1371/journal.pone.0299099 PMC10986986 38564618

[B17] Henson W. H. Mazzá C. Dall’Ara. E. (2023a). Deformable image registration based on single or multi-atlas methods for automatic muscle segmentation and the generation of augmented imaging datasets. PLOS ONE 18 (3), e0273446. 10.1371/journal.pone.0273446 36897869 PMC10004495

[B18] Hesamian M. H. Jia W. He X. Kennedy P. (2019). Deep learning techniques for medical image segmentation: achievements and challenges. J. Digital Imaging 32 (4), 582–596. 10.1007/s10278-019-00227-x PMC6646484 31144149

[B19] Ibtehaz N. Sohel Rahman M. (2020). MultiResUNet: rethinking the U-net architecture for multimodal biomedical image segmentation. Neural Netw. 121, 74–87. 10.1016/j.neunet.2019.08.025 31536901

[B20] Jackson P. T. Atapour-Abarghouei A. Bonner S. Breckon T. Obara B. (2019). Style augmentation: data augmentation via style randomization. arXiv.

[B21] Krizhevsky A. Sutskever I. Hinton G. E. (2017). ImageNet classification with deep convolutional neural networks. Commun. ACM 60 (6), 84–90. 10.1145/3065386

[B22] Krzysztofik M. Wilk M. Wojdała G. (2019). Maximizing muscle hypertrophy: a systematic review of advanced resistance training techniques and methods. Int. J. Environ. Res. Public Health 16 (24), 4897. 10.3390/ijerph16244897 31817252 PMC6950543

[B23] Kushnure D. T. Talbar S. N. (2021). MS-UNet: a multi-scale UNet with feature recalibration approach for automatic liver and tumor segmentation in CT images. Comput. Med. Imaging Graph. 89, 101885. 10.1016/j.compmedimag.2021.101885 33684731

[B24] Lafferty J. McCallum A. Pereira. F. (2001). “Conditional random fields: probabilistic models for segmenting and labeling sequence data,” in Icml '01: Proceedings of the Eighteenth International Conference on Machine Learning, Williams College, Williamstown, MA, June 28–July 1, 2001.

[B25] Lareau-Trudel E. Ghattas B. Jean P. Attarian S. Bendahan D. Salort-Campana E. (2015). Muscle quantitative MR imaging and clustering analysis in patients with facioscapulohumeral muscular dystrophy type 1. PLOS ONE 10 (7), e0132717. 10.1371/journal.pone.0132717 26181385 PMC4504465

[B26] Lisa D. (2023). Increased muscle fat infiltration is associated with reduced muscle strength in older women with obesity and dynapenia. arXiv.

[B27] Montefiori E. Kalkman B. M. Henson W. H. Paggiosi M. A. McCloskey E. V. Mazzà C. (2020). MRI-based anatomical characterisation of lower-limb muscles in older women. PLOS ONE 15 (12), e0242973. 10.1371/journal.pone.0242973 33259496 PMC7707470

[B28] Ni R. Meyer C. H. Blemker S. S. Hart J. M. Xue F. (2019). Automatic segmentation of all lower limb muscles from high-resolution magnetic resonance imaging using a cascaded three-dimensional deep convolutional neural network. J. Med. Imaging 6 (04), 1. 10.1117/1.JMI.6.4.044009 PMC6935014 31903406

[B29] Nishioka S. Yamanouchi A. Matsushita T. Nishioka E. Mori N. Taguchi S. (2021). Validity of calf circumference for estimating skeletal muscle mass for asian patients after stroke. Nutrition 82, 111028. 10.1016/j.nut.2020.111028 33139149

[B30] Oktay O. Jo S. Le Folgoc L. Lee M. Heinrich M. Misawa K. (2018). Attention U-net: learning where to look for the pancreas. arXiv.

[B31] Otsu N. (1979). A threshold selection method from gray-level histograms. IEEE Trans. Syst. Man, Cybern. 9 (1), 62–66. 10.1109/TSMC.1979.4310076

[B32] Pandy M. G. Andriacchi T. P. (2010). Muscle and joint function in human locomotion. Annu. Rev. Biomed. Eng. 12 (1), 401–433. 10.1146/annurev-bioeng-070909-105259 20617942

[B33] Ravikumar N. Ali G. Çimen S. Frangi A. F. Taylor Z. A. (2018). Group-wise similarity registration of point sets using student’s t-mixture model for statistical shape models. Med. Image Anal. 44, 156–176. 10.1016/j.media.2017.11.012 29248842

[B34] Ronneberger O. Fischer P. Brox T. (2015). U-net: convolutional networks for biomedical image segmentation. arXiv.

[B35] Shin H.-C. Tenenholtz N. A. Rogers J. K. Schwarz C. G. Senjem M. L. Gunter J. L. (2018). Medical image synthesis for data augmentation and anonymization using generative adversarial networks. arXiv.

[B36] Simonyan K. Zisserman A. (2015). Very deep convolutional networks for large-scale image recognition. arXiv.

[B37] Tao H. Li W. Qin X. Jia D. (2018). “Image semantic segmentation based on convolutional neural network and conditional random field,” in 2018 Tenth International Conference on Advanced Computational Intelligence (ICACI), Xiamen, China, 29-31 March 2018 (IEEE), 568–572. 10.1109/ICACI.2018.8377522

[B38] Thyreau B. Sato K. Fukuda H. Taki Y. (2018). Segmentation of the Hippocampus by transferring algorithmic knowledge for large cohort processing. Med. Image Anal. 43, 214–228. 10.1016/j.media.2017.11.004 29156419

[B39] Verdú-Díaz J. Nuñez-Peralta C. Tasca G. Vissing J. Straub V. Fernández-Torrón R. (2020). Accuracy of a machine learning muscle MRI-based tool for the diagnosis of muscular dystrophies. Neurology 94 (10), e1094–e1102. 10.1212/WNL.0000000000009068 32029545

[B40] Woo S. Park J. So K. (2018). CBAM: convolutional block attention module. arXiv.

[B41] Zhou Z. Rahman Siddiquee Md M. Tajbakhsh N. Liang J. (2018). UNet++: a nested U-net architecture for medical image segmentation. arXiv. 10.1007/978-3-030-00889-5_1 PMC7329239 32613207

[B42] Zhu J. Bolsterlee B. Brian V. Y. Cai C. Herbert R. D. Song Y. (2021). Deep learning methods for automatic segmentation of lower leg muscles and bones from MRI scans of children with and without cerebral palsy. NMR Biomed. 34 (12), e4609. 10.1002/nbm.4609 34545647

