# Supplementary material for: Automatic segmentation of skeletal muscles from MR images using modified U-Net and a novel data augmentation approach
Source: Front Bioeng Biotechnol. 2024 Feb 22;12:1355735. doi: 10.3389/fbioe.2024.1355735 (PMC10919285; doi:10.3389/fbioe.2024.1355735)
Supplement: Supplementary file 1 [file Table1.docx]

Supplementary Material

# Supplementary Tables

**Supplementary Table S1.** Model performance comparison under three metrics. The *p*-values in the table represent the significance results of the Wilcoxon signed-rank test conducted on muscles in all validation subjects between the models and Unet. The *p** values represent the significance results of the Wilcoxon signed-rank test conducted on muscles in all validation subjects across the cross-validation process between the models and Unet++. Significant differences are reported in bold ($p<0.05 or p* <0.05)$.

|  | DSC |  |  |  |  |
| --- | --- | --- | --- | --- | --- |
|  | Mean ±SD [%] | $p$ | Difference vs Unet [%] | $p^{*}$ | Difference vs Unet++ [%] |
| Unet | 0.810 ±0.108 | N/A | N/A | N/A | N/A |
| Unet++ | 0.811 ±0.098 | **0.026** | NS | N/A | N/A |
| AFFU | 0.828 ±0.079 | $\boldsymbol{<0.001}$ | 2.22 | $\boldsymbol{<0.001}$ | 2.10 |
| FFU | 0.826 ±0.087 | $\boldsymbol{<0.001}$ | 1.98 | $\boldsymbol{<0.001}$ | 1.85 |
|  | **1-RVE** |  |  |  |  |
|  | Mean ±SD [%] | $p$ | Difference vs Unet [%] | $p^{*}$ | Difference vs Unet++ [%] |
| Unet | 0.832 ±0.151 | N/A | N/A | N/A | N/A |
| Unet++ | 0.837 ±0.141 | 0.578 | NS | N/A | N/A |
| AFFU | 0.859 ±0.122 | **0.001** | 3.25 | $\boldsymbol{<0.001}$ | 2.63 |
| FFU | 0.853 ±0.123 | **0.002** | 2.52 | **0.006** | 1.91 |
|  | **HD** |  |  |  |  |
|  | Mean ±SD [mm] | $p$ | Difference vs Unet [%] | $p^{*}$ | Difference vs Unet++ [%] |
| Unet | 49.9 ±48.6 | N/A | N/A | N/A | N/A |
| Unet++ | 44.7 ±43.1 | 0.294 | NS | N/A | N/A |
| AFFU | 29.9 ±26.5 | $\boldsymbol{<0.001}$ | 40.1 | $\boldsymbol{<0.001}$ | 33.1 |
| FFU | 32.4 ±35.7 | $\boldsymbol{<0.001}$ | 35.1 | $\boldsymbol{<0.001}$ | 27.5 |

**Supplementary Table S2.** Average per-muscle performance of each model calculated from the cross-validation results. Each data recorded in the table represents the mean segmentation performance of each model for the same muscle across different validation samples. The values for the Hausdorff distance are rounded to one decimal place for improved readability.

|  | Unet | | | Unet++ | | | AFFU | | | FFU | | |
| --- | --- | --- | --- | --- | --- | --- | --- | --- | --- | --- | --- | --- |
|  | **DSC** | **1-RVE** | **HD** | **DSC** | **1-RVE** | **HD** | **DSC** | **1-RVE** | **HD** | **DSC** | **1-RVE** | **HD** |
| RF | 0.817 | 0.846 | 26.6 | 0.822 | 0.850 | 21.5 | 0.830 | 0.864 | 17.3 | 0.844 | 0.889 | 25.1 |
| VI | 0.809 | 0.910 | 47.5 | 0.817 | 0.924 | 31.5 | 0.825 | 0.907 | 29.1 | 0.825 | 0.900 | 34.0 |
| VL | 0.829 | 0.818 | 50.8 | 0.843 | 0.839 | 37.3 | 0.856 | 0.861 | 37.5 | 0.858 | 0.865 | 34.0 |
| VM | 0.848 | 0.833 | 55.2 | 0.851 | 0.829 | 35.6 | 0.872 | 0.858 | 30.6 | 0.874 | 0.862 | 43.4 |
| SAT | 0.788 | 0.782 | 43.6 | 0.789 | 0.788 | 43.1 | 0.822 | 0.840 | 16.0 | 0.817 | 0.833 | 16.9 |
| SMB | 0.808 | 0.814 | 51.7 | 0.807 | 0.853 | 43.4 | 0.819 | 0.866 | 48.2 | 0.811 | 0.843 | 38.8 |
| SMT | 0.776 | 0.789 | 47.4 | 0.785 | 0.785 | 37.1 | 0.804 | 0.812 | 35.7 | 0.798 | 0.806 | 33.6 |
| GRA | 0.718 | 0.768 | 22.0 | 0.719 | 0.766 | 23.3 | 0.752 | 0.853 | 17.8 | 0.728 | 0.792 | 17.4 |
| BCB | 0.800 | 0.775 | 83.2 | 0.785 | 0.758 | 74.5 | 0.809 | 0.791 | 23.9 | 0.815 | 0.789 | 35.8 |
| BCL | 0.811 | 0.832 | 62.1 | 0.818 | 0.829 | 42.0 | 0.847 | 0.859 | 25.7 | 0.845 | 0.859 | 52.0 |
| AM | 0.850 | 0.877 | 58.9 | 0.857 | 0.899 | 43.3 | 0.869 | 0.901 | 34.7 | 0.866 | 0.893 | 37.5 |
| AB | 0.753 | 0.833 | 34.4 | 0.753 | 0.858 | 41.3 | 0.761 | 0.848 | 21.4 | 0.759 | 0.838 | 18.1 |
| AL | 0.812 | 0.847 | 26.2 | 0.809 | 0.847 | 27.1 | 0.823 | 0.855 | 18.1 | 0.828 | 0.867 | 30.2 |
| GM | 0.901 | 0.882 | 69.3 | 0.900 | 0.884 | 60.1 | 0.907 | 0.906 | 61.1 | 0.908 | 0.900 | 44.4 |
| IL | 0.843 | 0.879 | 67.6 | 0.837 | 0.879 | 81.8 | 0.859 | 0.899 | 35.2 | 0.859 | 0.894 | 31.7 |
| TFL | 0.798 | 0.834 | 52.7 | 0.783 | 0.807 | 72.2 | 0.791 | 0.816 | 25.3 | 0.788 | 0.821 | 25.4 |

**Supplementary Table S3.** Average model performance comparison including all test subjects.

|  | DSC |  |  |  |  |
| --- | --- | --- | --- | --- | --- |
|  | Mean ±SD [%] | $p$ | Difference vs Unet [%] | $p^{*}$ | Difference vs Unet++ [%] |
| Unet | 0.843 ±0.062 | N/A | N/A | N/A | N/A |
| Unet++ | 0.837 ±0.067 | $\boldsymbol{<0.001}$ | -0.70 | N/A | N/A |
| AFFU | 0.848 ±0.058 | $\boldsymbol{<0.001}$ | 0.59 | $\boldsymbol{<0.001}$ | 1.31 |
| FFU | 0.848 ±0.058 | $\boldsymbol{<0.001}$ | 0.59 | $\boldsymbol{<0.001}$ | 1.31 |
|  | **1-RVE** |  |  |  |  |
|  | Mean ±SD [%] | $p$ | Difference vs Unet [%] | $p^{*}$ | Difference vs Unet++ [%] |
| Unet | 0.893 ±0.097 | N/A | N/A | N/A | N/A |
| Unet++ | 0.884 ±0.103 | $\boldsymbol{<0.001}$ | -1.01 | N/A | N/A |
| AFFU | 0.894 ±0.096 | 0.674 | NS | **0.014** | 1.13 |
| FFU | 0.894 ±0.095 | 0.686 | NS | **0.011** | 1.13 |
|  | **HD** |  |  |  |  |
|  | Mean ±SD [mm] | $p$ | Difference vs Unet [%] | $p^{*}$ | Difference vs Unet++ [%] |
| Unet | 49.1 ±52.8 | N/A | N/A | N/A | N/A |
| Unet++ | 51.7 ±49.5 | 0.242 | NS | N/A | N/A |
| AFFU | 30.2 ±38.7 | $\boldsymbol{<0.001}$ | 38.5 | $\boldsymbol{<0.001}$ | 41.6 |
| FFU | 35.9 ±52.5 | $\boldsymbol{<0.001}$ | 26.9 | $\boldsymbol{<0.001}$ | 30.6 |

**Supplementary Table S4.** Average per-muscle performance of each model calculated from all test subjects. The data in the table represent the average segmentation performance of the same muscle for each model across all test scans for part2.

|  | Unet | | | Unet++ | | | AFFU | | | FFU | | |
| --- | --- | --- | --- | --- | --- | --- | --- | --- | --- | --- | --- | --- |
|  | **DSC** | **1-RVE** | **HD** | **DSC** | **1-RVE** | **HD** | **DSC** | **1-RVE** | **HD** | **DSC** | **1-RVE** | **HD** |
| RF | 0.869 | 0.933 | 21.2 | 0.855 | 0.921 | 30.7 | 0.883 | 0.947 | 18.1 | 0.881 | 0.947 | 22.2 |
| VI | 0.791 | 0.813 | 45.7 | 0.787 | 0.816 | 32.6 | 0.796 | 0.805 | 38.4 | 0.790 | 0.799 | 29.5 |
| VL | 0.842 | 0.916 | 72.2 | 0.838 | 0.912 | 51.4 | 0.846 | 0.903 | 49.6 | 0.837 | 0.893 | 66.1 |
| VM | 0.882 | 0.910 | 57.2 | 0.877 | 0.895 | 32.9 | 0.887 | 0.915 | 37.7 | 0.887 | 0.907 | 38.2 |
| SAT | 0.822 | 0.908 | 41.5 | 0.821 | 0.901 | 55.5 | 0.840 | 0.890 | 21.9 | 0.840 | 0.903 | 25.0 |
| SMB | 0.844 | 0.886 | 62.3 | 0.832 | 0.869 | 46.1 | 0.844 | 0.866 | 33.0 | 0.843 | 0.865 | 29.3 |
| SMT | 0.836 | 0.906 | 37.1 | 0.833 | 0.902 | 37.7 | 0.840 | 0.918 | 31.6 | 0.841 | 0.918 | 45.3 |
| GRA | 0.832 | 0.895 | 30.0 | 0.831 | 0.871 | 29.3 | 0.840 | 0.900 | 21.4 | 0.842 | 0.900 | 24.2 |
| BCB | 0.812 | 0.862 | 35.0 | 0.804 | 0.857 | 90.2 | 0.816 | 0.867 | 19.9 | 0.821 | 0.866 | 33.5 |
| BCL | 0.885 | 0.911 | 37.5 | 0.876 | 0.910 | 57.8 | 0.886 | 0.906 | 26.1 | 0.887 | 0.909 | 49.9 |
| AM | 0.884 | 0.918 | 69.8 | 0.877 | 0.903 | 53.7 | 0.886 | 0.916 | 48.8 | 0.886 | 0.919 | 40.7 |
| AB | 0.775 | 0.827 | 32.0 | 0.767 | 0.840 | 44.8 | 0.770 | 0.830 | 17.0 | 0.770 | 0.816 | 17.9 |
| AL | 0.835 | 0.920 | 40.1 | 0.821 | 0.897 | 49.5 | 0.843 | 0.921 | 17.1 | 0.843 | 0.919 | 17.0 |
| GM | 0.919 | 0.928 | 72.9 | 0.920 | 0.927 | 62.1 | 0.925 | 0.957 | 65.3 | 0.925 | 0.964 | 90.4 |
| IL | 0.842 | 0.874 | 78.8 | 0.839 | 0.866 | 101.7 | 0.859 | 0.900 | 20.6 | 0.857 | 0.902 | 31.5 |
| TFL | 0.824 | 0.878 | 52.3 | 0.817 | 0.860 | 51.3 | 0.810 | 0.858 | 17.2 | 0.812 | 0.870 | 14.3 |

**Supplementary Table S5.** Average performance of each model for PMW-2 and PMW-OB. The p-values in the table represent the significance results of the Wilcoxon signed-rank test conducted on muscles in all validation subjects between the models and Unet or Unet++ about two different cohorts PMW-2 and PMW-OB. Significant differences are reported in bold ($p<0.05 or p* <0.05)$.

|  |  | PMW-2 |  |  |  |  | PMW-OB |  |  |  |
| --- | --- | --- | --- | --- | --- | --- | --- | --- | --- | --- |
|  | **DSC** |  |  |  |  |  |  |  |  |  |
|  | Mean ±SD [%] | $p$ | Difference vs Unet [%] | $p^{*}$ | Difference vs Unet++ [%] | Mean ±SD [%] | $p$ | Difference vs Unet [%] | $p^{*}$ | Difference vs Unet++ [%] |
| Unet | 0.828 ±0.073 | N/A | N/A | N/A | N/A | 0.857 ±0.049 | N/A | N/A | N/A | N/A |
| Unet++ | 0.820 ±0.080 | $\boldsymbol{<0.001}$ | -0.97 | N/A | N/A | 0.853 ±0.051 | $\boldsymbol{<0.001}$ | -0.47 | N/A | N/A |
| AFFU | 0.833 ±0.065 | **0.008** | 0.60 | $\boldsymbol{<0.001}$ | 1.58 | 0.862 ±0.048 | $\boldsymbol{<0.001}$ | 0.58 | $\boldsymbol{<0.001}$ | 1.06 |
| FFU | 0.834 ±0.065 | **0.006** | 0.72 | 0.743 | NS | 0.861 ±0.049 | **0.006** | 0.47 | $\boldsymbol{<0.001}$ | 0.94 |
|  | **1-RVE** |  |  |  |  |  |  |  |  |  |
|  | Mean ±SD [%] | $p$ | Difference vs Unet [%] | $p^{*}$ | Difference vs Unet++ [%] | Mean ±SD [%] | $p$ | Difference vs Unet [%] | $p^{*}$ | Difference vs Unet++ [%] |
| Unet | 0.880 ±0.105 | N/A | N/A | N/A | N/A | 0.914 ±0.072 | N/A | N/A | N/A | N/A |
| Unet++ | 0.868 ±0.116 | $\boldsymbol{<0.001}$ | -1.36 | N/A | N/A | 0.908 ±0.073 | **0.003** | -0.66 | N/A | N/A |
| AFFU | 0.873 ±0.105 | 0.470 | NS | 0.264 | NS | 0.919 ±0.076 | 0.242 | NS | **0.021** | 1.21 |
| FFU | 0.876 ±0.105 | 0.737 | NS | 0.166 | NS | 0.917 ±0.075 | 0.484 | NS | **0.046** | 0.99 |
|  | **HD** |  |  |  |  |  |  |  |  |  |
|  | Mean ±SD [mm] | $p$ | Difference vs Unet [%] | $p^{*}$ | Difference vs Unet++ [%] | Mean ±SD [mm] | $p$ | Difference vs Unet [%] | $p^{*}$ | Difference vs Unet++ [%] |
| Unet | 45.0 ±47.6 | N/A | N/A | N/A | N/A | 54.5 ±58.4 | N/A | N/A | N/A | N/A |
| Unet++ | 47.3 ±44.1 | 0.606 | NS | N/A | N/A | 57.4 ±54.8 | 0.446 | NS | N/A | N/A |
| AFFU | 25.9 ±27.9 | $\boldsymbol{<0.001}$ | 42.4 | $\boldsymbol{<0.001}$ | 45.2 | 34.8 ±46.8 | $\boldsymbol{<0.001}$ | 36.1 | $\boldsymbol{<0.001}$ | 39.4 |
| FFU | 29.8 ±32.2 | $\boldsymbol{<0.001}$ | 33.8 | $\boldsymbol{<0.001}$ | 40.0 | 42.5 ±65.7 | $\boldsymbol{<0.001}$ | 22.0 | $\boldsymbol{<0.001}$ | 26.0 |

**Supplementary Table S6.** Average per-muscle performance of each model calculated from eight healthy old women.

| PMW-2 | | | | | | | | | | | | |
| --- | --- | --- | --- | --- | --- | --- | --- | --- | --- | --- | --- | --- |
|  | Unet | | | Unet++ | | | AFFU | | | FFU | | |
|  | **DSC** | **1-RVE** | **HD** | **DSC** | **1-RVE** | **HD** | **DSC** | **1-RVE** | **HD** | **DSC** | **1-RVE** | **HD** |
| RF | 0.825 | 0.942 | 23.6 | 0.800 | 0.921 | 31.7 | 0.852 | 0.939 | 18.8 | 0.846 | 0.949 | 19.1 |
| VI | 0.780 | 0.845 | 34.3 | 0.775 | 0.844 | 32.8 | 0.780 | 0.856 | 31.7 | 0.773 | 0.845 | 32.4 |
| VL | 0.826 | 0.893 | 61.5 | 0.819 | 0.890 | 41.0 | 0.827 | 0.891 | 32.6 | 0.817 | 0.879 | 50.2 |
| VM | 0.861 | 0.858 | 34.6 | 0.856 | 0.841 | 35.5 | 0.865 | 0.862 | 21.2 | 0.867 | 0.851 | 21.9 |
| SAT | 0.796 | 0.899 | 33.5 | 0.801 | 0.888 | 41.8 | 0.813 | 0.854 | 19.2 | 0.817 | 0.870 | 21.8 |
| SMB | 0.817 | 0.834 | 71.3 | 0.793 | 0.792 | 36.5 | 0.830 | 0.854 | 22.1 | 0.827 | 0.846 | 26.9 |
| SMT | 0.829 | 0.903 | 38.4 | 0.822 | 0.894 | 27.9 | 0.823 | 0.919 | 29.5 | 0.828 | 0.925 | 29.8 |
| GRA | 0.830 | 0.935 | 24.9 | 0.831 | 0.917 | 24.4 | 0.828 | 0.874 | 20.5 | 0.836 | 0.889 | 17.9 |
| BCB | 0.788 | 0.819 | 34.3 | 0.775 | 0.795 | 93.4 | 0.788 | 0.806 | 22.0 | 0.798 | 0.810 | 22.0 |
| BCL | 0.874 | 0.909 | 45.3 | 0.863 | 0.900 | 60.4 | 0.870 | 0.885 | 22.8 | 0.874 | 0.897 | 29.7 |
| AM | 0.872 | 0.860 | 63.3 | 0.860 | 0.829 | 51.0 | 0.873 | 0.870 | 48.2 | 0.873 | 0.871 | 46.7 |
| AB | 0.774 | 0.771 | 35.5 | 0.770 | 0.781 | 49.5 | 0.767 | 0.783 | 16.1 | 0.772 | 0.773 | 16.6 |
| AL | 0.831 | 0.920 | 34.6 | 0.823 | 0.901 | 32.6 | 0.840 | 0.888 | 19.4 | 0.842 | 0.901 | 18.4 |
| GM | 0.920 | 0.943 | 68.8 | 0.920 | 0.945 | 52.7 | 0.925 | 0.968 | 54.4 | 0.924 | 0.971 | 72.2 |
| IL | 0.823 | 0.880 | 87.5 | 0.822 | 0.890 | 112.9 | 0.849 | 0.910 | 18.6 | 0.845 | 0.911 | 37.3 |
| TFL | 0.806 | 0.865 | 29.2 | 0.797 | 0.859 | 31.9 | 0.796 | 0.812 | 16.9 | 0.799 | 0.837 | 14.4 |

**Supplementary Table S7.** Average per-muscle performance of each model calculated from ten old obese women. The data in Tables S6/S7 represent the average performance evaluation results of each muscle for each model across different cohorts, PMW-2, and PMW-OB.

| PMW-OB | | | | | | | | | | | | |
| --- | --- | --- | --- | --- | --- | --- | --- | --- | --- | --- | --- | --- |
|  | Unet | | | Unet++ | | | AFFU | | | FFU | | |
|  | **DSC** | **1-RVE** | **HD** | **DSC** | **1-RVE** | **HD** | **DSC** | **1-RVE** | **HD** | **DSC** | **1-RVE** | **HD** |
| RF | 0.903 | 0.924 | 20.3 | 0.897 | 0.917 | 31.9 | 0.911 | 0.959 | 18.0 | 0.911 | 0.949 | 25.7 |
| VI | 0.796 | 0.778 | 55.8 | 0.794 | 0.783 | 29.9 | 0.803 | 0.758 | 44.9 | 0.799 | 0.757 | 26.8 |
| VL | 0.855 | 0.957 | 84.2 | 0.853 | 0.953 | 61.1 | 0.861 | 0.926 | 65.3 | 0.853 | 0.919 | 83.1 |
| VM | 0.899 | 0.954 | 77.5 | 0.893 | 0.941 | 31.3 | 0.903 | 0.960 | 52.7 | 0.903 | 0.953 | 53.2 |
| SAT | 0.847 | 0.939 | 49.7 | 0.842 | 0.933 | 69.4 | 0.863 | 0.935 | 24.1 | 0.862 | 0.947 | 28.2 |
| SMB | 0.866 | 0.930 | 57.5 | 0.866 | 0.934 | 53.6 | 0.859 | 0.872 | 42.3 | 0.859 | 0.875 | 31.5 |
| SMT | 0.844 | 0.941 | 38.1 | 0.845 | 0.944 | 47.5 | 0.854 | 0.946 | 35.0 | 0.854 | 0.943 | 60.6 |
| GRA | 0.845 | 0.904 | 34.5 | 0.843 | 0.880 | 33.5 | 0.854 | 0.928 | 22.0 | 0.854 | 0.923 | 30.1 |
| BCB | 0.832 | 0.924 | 37.8 | 0.828 | 0.936 | 95.5 | 0.839 | 0.945 | 19.2 | 0.842 | 0.939 | 45.0 |
| BCL | 0.895 | 0.925 | 33.6 | 0.886 | 0.931 | 60.3 | 0.898 | 0.935 | 30.8 | 0.897 | 0.931 | 70.3 |
| AM | 0.899 | 0.965 | 78.8 | 0.896 | 0.963 | 58.0 | 0.900 | 0.958 | 50.8 | 0.899 | 0.962 | 37.1 |
| AB | 0.777 | 0.877 | 31.3 | 0.766 | 0.882 | 44.2 | 0.774 | 0.878 | 18.3 | 0.770 | 0.858 | 19.8 |
| AL | 0.835 | 0.916 | 46.8 | 0.817 | 0.887 | 66.3 | 0.842 | 0.945 | 15.2 | 0.841 | 0.932 | 15.6 |
| GM | 0.923 | 0.929 | 78.8 | 0.923 | 0.928 | 69.8 | 0.928 | 0.962 | 78.8 | 0.929 | 0.971 | 112.7 |
| IL | 0.854 | 0.873 | 75.2 | 0.850 | 0.852 | 97.4 | 0.865 | 0.894 | 22.4 | 0.865 | 0.897 | 27.6 |
| TFL | 0.848 | 0.884 | 72.7 | 0.842 | 0.858 | 68.7 | 0.834 | 0.906 | 16.9 | 0.835 | 0.909 | 13.1 |

**Supplementary Table S8.** Average per-muscle performance of Unet and AFFU calculated with and without augmentation data on all test scans. The results for each muscle represent the average values obtained from predictions on all test scans using the same model.

|  | Unet | | | | | | | | AFFU | | | | | | | |
| --- | --- | --- | --- | --- | --- | --- | --- | --- | --- | --- | --- | --- | --- | --- | --- | --- |
|  | **DSC** | | **1-RVE** | | | **HD** | | | **DSC** | | **1-RVE** | | | **HD** | | |
|  | Without aug. | With aug. | | Without aug. | With aug. | | Without aug. | With aug. | Without aug. | With aug. | | Without aug. | With aug. | | Without aug. | With aug. |
| RF | 0.869 | 0.888 | | 0.933 | 0.930 | | 21.2 | 18.3 | 0.883 | 0.892 | | 0.947 | 0.936 | | 18.1 | 16.7 |
| VI | 0.791 | 0.800 | | 0.813 | 0.814 | | 45.7 | 52.8 | 0.796 | 0.797 | | 0.805 | 0.792 | | 38.4 | 27.3 |
| VL | 0.842 | 0.851 | | 0.916 | 0.916 | | 72.2 | 60.8 | 0.846 | 0.844 | | 0.903 | 0.909 | | 49.6 | 48.0 |
| VM | 0.882 | 0.893 | | 0.910 | 0.909 | | 57.2 | 39.1 | 0.887 | 0.894 | | 0.915 | 0.915 | | 37.7 | 31.1 |
| SAT | 0.822 | 0.843 | | 0.908 | 0.897 | | 41.5 | 38.2 | 0.840 | 0.846 | | 0.890 | 0.903 | | 21.9 | 20.9 |
| SMB | 0.844 | 0.864 | | 0.886 | 0.919 | | 62.3 | 35.4 | 0.844 | 0.857 | | 0.866 | 0.897 | | 33.0 | 27.4 |
| SMT | 0.836 | 0.855 | | 0.906 | 0.921 | | 37.1 | 42.1 | 0.840 | 0.853 | | 0.918 | 0.913 | | 31.6 | 31.2 |
| GRA | 0.832 | 0.845 | | 0.895 | 0.920 | | 30.0 | 23.2 | 0.840 | 0.843 | | 0.900 | 0.914 | | 21.4 | 20.1 |
| BCB | 0.812 | 0.824 | | 0.862 | 0.855 | | 35.0 | 35.4 | 0.816 | 0.824 | | 0.867 | 0.865 | | 19.9 | 19.4 |
| BCL | 0.885 | 0.892 | | 0.911 | 0.909 | | 37.5 | 44.1 | 0.886 | 0.894 | | 0.906 | 0.919 | | 26.1 | 24.0 |
| AM | 0.884 | 0.890 | | 0.918 | 0.913 | | 69.8 | 75.3 | 0.886 | 0.886 | | 0.916 | 0.916 | | 48.8 | 42.6 |
| AB | 0.775 | 0.787 | | 0.827 | 0.855 | | 32.0 | 22.4 | 0.770 | 0.770 | | 0.830 | 0.778 | | 17.0 | 16.4 |
| AL | 0.835 | 0.851 | | 0.920 | 0.939 | | 40.1 | 18.2 | 0.843 | 0.843 | | 0.921 | 0.937 | | 17.1 | 17.4 |
| GM | 0.919 | 0.925 | | 0.928 | 0.936 | | 72.9 | 92.8 | 0.925 | 0.925 | | 0.957 | 0.954 | | 65.3 | 96.6 |
| IL | 0.842 | 0.852 | | 0.874 | 0.898 | | 78.8 | 62.9 | 0.859 | 0.858 | | 0.900 | 0.907 | | 20.6 | 39.4 |
| TFL | 0.824 | 0.836 | | 0.878 | 0.877 | | 52.3 | 35.5 | 0.810 | 0.821 | | 0.858 | 0.892 | | 17.2 | 22.6 |

**Supplementary Table S9.** Average per-muscle performance of Unet and AFFU with/without augmentation data on one post-menopausal woman. Since only one test scan was included, each data point in above table represents the mean value of three repetitions from the same test subject.

|  | Unet | | | | | | AFFU | | | | | |
| --- | --- | --- | --- | --- | --- | --- | --- | --- | --- | --- | --- | --- |
| PMW-1 | | | | | | | | | | | | |
|  | **DSC** | | **1-RVE** | | **HD** | | **DSC** | | **1-RVE** | | **HD** | |
|  | Without aug. | With aug. | Without aug. | With aug. | Without aug. | With aug. | Without aug. | With aug. | Without aug. | With aug. | Without aug. | With aug. |
| RF | 0.874 | 0.891 | 0.955 | 0.937 | 11.9 | 10.7 | 0.854 | 0.887 | 0.882 | 0.985 | 13.8 | 12.0 |
| VI | 0.817 | 0.820 | 0.900 | 0.922 | 35.3 | 39.8 | 0.853 | 0.853 | 0.861 | 0.866 | 27.9 | 21.5 |
| VL | 0.834 | 0.843 | 0.678 | 0.690 | 37.6 | 25.7 | 0.856 | 0.863 | 0.759 | 0.755 | 28.4 | 25.8 |
| VM | 0.880 | 0.899 | 0.880 | 0.865 | 35.1 | 25.9 | 0.898 | 0.899 | 0.893 | 0.884 | 19.3 | 15.9 |
| SAT | 0.786 | 0.790 | 0.660 | 0.556 | 24.7 | 21.2 | 0.821 | 0.798 | 0.731 | 0.621 | 21.6 | 16.0 |
| SMB | 0.837 | 0.839 | 0.869 | 0.895 | 38.3 | 33.4 | 0.810 | 0.817 | 0.903 | 0.938 | 27.8 | 24.0 |
| SMT | 0.811 | 0.804 | 0.585 | 0.572 | 16.7 | 16.3 | 0.820 | 0.813 | 0.624 | 0.632 | 14.5 | 14.0 |
| GRA | 0.725 | 0.746 | 0.495 | 0.468 | 26.1 | 25.0 | 0.791 | 0.781 | 0.834 | 0.727 | 21.6 | 21.3 |
| BCB | 0.799 | 0.804 | 0.585 | 0.613 | 12.6 | 22.6 | 0.802 | 0.806 | 0.563 | 0.602 | 10.9 | 11.5 |
| BCL | 0.881 | 0.884 | 0.782 | 0.760 | 13.4 | 11.6 | 0.892 | 0.884 | 0.794 | 0.767 | 6.3 | 6.2 |
| AM | 0.836 | 0.853 | 0.901 | 0.863 | 31.3 | 32.2 | 0.853 | 0.856 | 0.861 | 0.863 | 34.1 | 32.7 |
| AB | 0.764 | 0.777 | 0.774 | 0.820 | 11.6 | 9.6 | 0.758 | 0.750 | 0.727 | 0.697 | 11.4 | 10.1 |
| AL | 0.858 | 0.867 | 0.963 | 0.948 | 17.4 | 13.9 | 0.870 | 0.859 | 0.941 | 0.990 | 17.4 | 13.2 |
| GM | 0.881 | 0.888 | 0.787 | 0.806 | 47.3 | 16.0 | 0.893 | 0.893 | 0.822 | 0.829 | 17.5 | 14.4 |
| IL | 0.866 | 0.857 | 0.834 | 0.813 | 43.9 | 32.6 | 0.879 | 0.874 | 0.870 | 0.853 | 19.5 | 25.9 |
| TFL | 0.738 | 0.782 | 0.927 | 0.960 | 32.6 | 21.1 | 0.686 | 0.743 | 0.747 | 0.890 | 23.4 | 19.7 |

**Supplementary Table S10.** Average per-muscle performance of Unet and AFFU with/without augmentation data on ten old obese women. Each data point represents the mean performance of each muscle for a particular model across all individuals within the same cohort.

|  | Unet | | | | | | AFFU | | | | | |
| --- | --- | --- | --- | --- | --- | --- | --- | --- | --- | --- | --- | --- |
| PMW-OB | | | | | | | | | | | | |
|  | **DSC** | | **1-RVE** | | **HD** | | **DSC** | | **1-RVE** | | **HD** | |
|  | Without aug. | With aug. | Without aug. | With aug. | Without aug. | With aug. | Without aug. | With aug. | Without aug. | With aug. | Without aug. | With aug. |
| RF | 0.903 | 0.911 | 0.924 | 0.925 | 20.3 | 18.0 | 0.911 | 0.912 | 0.959 | 0.941 | 18.0 | 16.4 |
| VI | 0.796 | 0.806 | 0.778 | 0.775 | 55.8 | 69.1 | 0.803 | 0.804 | 0.758 | 0.754 | 44.9 | 27.3 |
| VL | 0.855 | 0.862 | 0.957 | 0.945 | 84.2 | 69.9 | 0.861 | 0.856 | 0.926 | 0.926 | 65.3 | 60.3 |
| VM | 0.899 | 0.905 | 0.954 | 0.947 | 77.5 | 51.1 | 0.903 | 0.910 | 0.960 | 0.954 | 52.7 | 38.5 |
| SAT | 0.847 | 0.864 | 0.939 | 0.928 | 49.7 | 50.2 | 0.863 | 0.867 | 0.935 | 0.950 | 24.1 | 24.9 |
| SMB | 0.866 | 0.875 | 0.930 | 0.940 | 57.5 | 39.9 | 0.859 | 0.870 | 0.872 | 0.905 | 42.3 | 32.7 |
| SMT | 0.844 | 0.863 | 0.941 | 0.951 | 38.1 | 44.9 | 0.854 | 0.863 | 0.946 | 0.935 | 35.0 | 33.7 |
| GRA | 0.845 | 0.858 | 0.904 | 0.942 | 34.5 | 28.0 | 0.854 | 0.850 | 0.928 | 0.930 | 22.0 | 19.0 |
| BCB | 0.832 | 0.838 | 0.924 | 0.901 | 37.8 | 35.3 | 0.839 | 0.847 | 0.945 | 0.919 | 19.2 | 17.1 |
| BCL | 0.895 | 0.897 | 0.925 | 0.917 | 33.6 | 63.6 | 0.898 | 0.901 | 0.935 | 0.938 | 30.8 | 27.5 |
| AM | 0.899 | 0.903 | 0.965 | 0.960 | 78.8 | 77.3 | 0.900 | 0.896 | 0.958 | 0.951 | 50.8 | 40.2 |
| AB | 0.777 | 0.787 | 0.877 | 0.888 | 31.3 | 21.5 | 0.774 | 0.771 | 0.878 | 0.791 | 18.3 | 17.3 |
| AL | 0.835 | 0.847 | 0.916 | 0.937 | 46.8 | 20.7 | 0.842 | 0.840 | 0.945 | 0.951 | 15.2 | 18.7 |
| GM | 0.923 | 0.928 | 0.929 | 0.936 | 78.8 | 114.8 | 0.928 | 0.929 | 0.962 | 0.958 | 78.8 | 137.7 |
| IL | 0.854 | 0.861 | 0.873 | 0.893 | 75.2 | 67.3 | 0.865 | 0.864 | 0.894 | 0.896 | 22.4 | 38.6 |
| TFL | 0.848 | 0.855 | 0.884 | 0.868 | 72.7 | 53.3 | 0.834 | 0.838 | 0.906 | 0.911 | 16.9 | 28.6 |

**Supplementary Table S11.** Average per-muscle performance of Unet and AFFU with/without augmentation data on eight old healthy women. Each data point represents the mean performance of each muscle for a particular model across all individuals within the same cohort.

|  | Unet | | | | | | AFFU | | | | | |
| --- | --- | --- | --- | --- | --- | --- | --- | --- | --- | --- | --- | --- |
| PMW-2 | | | | | | | | | | | | |
|  | **DSC** | | **1-RVE** | | **HD** | | **DSC** | | **1-RVE** | | **HD** | |
|  | Without aug. | With aug. | Without aug. | With aug. | Without aug. | With aug. | Without aug. | With aug. | Without aug. | With aug. | Without aug. | With aug. |
| RF | 0.825 | 0.858 | 0.942 | 0.935 | 23.6 | 19.7 | 0.852 | 0.868 | 0.939 | 0.925 | 18.8 | 17.6 |
| VI | 0.780 | 0.790 | 0.845 | 0.849 | 34.3 | 34.1 | 0.780 | 0.782 | 0.856 | 0.831 | 31.7 | 27.9 |
| VL | 0.826 | 0.838 | 0.893 | 0.908 | 61.5 | 53.8 | 0.827 | 0.826 | 0.891 | 0.907 | 32.6 | 35.3 |
| VM | 0.861 | 0.876 | 0.858 | 0.868 | 34.6 | 25.7 | 0.865 | 0.874 | 0.862 | 0.870 | 21.2 | 23.7 |
| SAT | 0.796 | 0.822 | 0.899 | 0.902 | 33.5 | 25.5 | 0.813 | 0.824 | 0.854 | 0.879 | 19.2 | 16.6 |
| SMB | 0.817 | 0.852 | 0.834 | 0.896 | 71.3 | 30.0 | 0.830 | 0.846 | 0.854 | 0.881 | 22.1 | 21.2 |
| SMT | 0.829 | 0.852 | 0.903 | 0.926 | 38.4 | 41.8 | 0.823 | 0.846 | 0.919 | 0.920 | 29.5 | 30.2 |
| GRA | 0.830 | 0.842 | 0.935 | 0.950 | 24.9 | 16.9 | 0.828 | 0.841 | 0.874 | 0.916 | 20.5 | 21.3 |
| BCB | 0.788 | 0.810 | 0.819 | 0.828 | 34.3 | 37.2 | 0.788 | 0.797 | 0.806 | 0.830 | 22.0 | 23.3 |
| BCL | 0.874 | 0.886 | 0.909 | 0.919 | 45.3 | 23.7 | 0.870 | 0.888 | 0.885 | 0.914 | 22.8 | 21.9 |
| AM | 0.872 | 0.879 | 0.860 | 0.861 | 63.3 | 78.2 | 0.873 | 0.878 | 0.870 | 0.877 | 48.2 | 46.9 |
| AB | 0.774 | 0.789 | 0.771 | 0.818 | 35.5 | 25.0 | 0.767 | 0.770 | 0.783 | 0.771 | 16.1 | 16.1 |
| AL | 0.831 | 0.852 | 0.920 | 0.941 | 34.6 | 15.6 | 0.840 | 0.844 | 0.888 | 0.912 | 19.4 | 16.3 |
| GM | 0.920 | 0.926 | 0.943 | 0.952 | 68.8 | 74.8 | 0.925 | 0.924 | 0.968 | 0.965 | 54.4 | 55.4 |
| IL | 0.823 | 0.840 | 0.880 | 0.915 | 87.5 | 61.2 | 0.849 | 0.849 | 0.910 | 0.926 | 18.6 | 42.1 |
| TFL | 0.806 | 0.820 | 0.865 | 0.878 | 29.2 | 15.0 | 0.796 | 0.810 | 0.812 | 0.869 | 16.9 | 15.5 |
